# Transcriptome analysis reveals differentially expressed genes associated with high rates of egg production in chicken hypothalamic-pituitary-ovarian axis

**DOI:** 10.1038/s41598-020-62886-z

**Published:** 2020-04-06

**Authors:** Shailendra Kumar Mishra, Binlong Chen, Qing Zhu, Zhongxian Xu, Chunyou Ning, Huadong Yin, Yan Wang, Xiaoling Zhao, Xiaolan Fan, Mingyao Yang, Deying Yang, Qingyong Ni, Yan Li, Mingwang Zhang, Diyan Li

**Affiliations:** 1 Farm Animal Genetic Resources Exploration and Innovation Key Laboratory of Sichuan Province, Sichuan Agricultural University, Chengdu, 611130 China; 2 XiChang University, Xuefu road No 1, Anning town, Xichang, Liangshan prefecture, Sichuan province, China

**Keywords:** Animal breeding, Transcriptional regulatory elements

## Abstract

The hypothalamic-pituitary-ovarian (HPO) axis regulates the breeding process cycle of laying hens. However, the key regulatory genes of the HPO axis and pathways that drive chicken egg laying performance remain elusive. A total of 856 Chinese Luhua chicken was raised and the highest two hundred and the lowest two hundred chicken egg production were considered as high egg production (HEP) and low egg production (LEP) according to the total egg number at 300 days of age, respectively. RNA-seq sequencing (RNA-Seq) was conducted to explore the chicken transcriptome from the hypothalamus, pituitary gland and ovary tissue of 6 Chinese Luhua chicken with 3 high and low-rate egg production. In total, 76.09 Gb RNA-seq sequences were generated from 15 libraries with an average of 5.07 Gb for each library. Further analysis showed that 414, 356 and 10 differentially expressed genes (DEGs) were identified in pituitary gland, ovary and hypothalamus between HEP and LEP chickens, respectively. In pituitary gland, DEGs were involve in regulation of cellular glucose homeostasis, Ras protein signal transduction, negative regulation of hormone secretion. In Ovary DEGs were mainly involved in embryonic organ development, regulation of canonical Wnt signaling, response to peptide hormone. Our study identified DEGs that regulate mTOR signaling pathway, Jak-STAT signaling pathway, Tryptophan metabolism and PI3K-Akt signaling pathways at HPO-axis in laying hens. These important data contribute to improve our understanding of reproductive biology of chicken and isolating effective molecular markers that can be used for genetic selection in Chinese domestic Luhua chicken.

## Introduction

Traditional breeding strategies aimed to improve the chicken egg production are based on long term selection of egg number and laying rate, which are often laborious and time consuming^1^. For practical breeding, quality chickens were primitively produced by indigenous chicken breeds, which are generally low egg production and slowly-growing with poor feed conversion. In certain regions of the world, there is a growing demand for slow-growing and colour-feathered quality chickens. Among them, the ‘Label Rouge’ in France and ‘Three Yellow’ in China are two famous examples. In terms of nutrition and management of chickens, rapid genetic selection can improve economic efficiency. Currently, the breeding for quality chickens in China is characterized with crossbreeding between native breeds and highly-selected lines with relatively high egg production or rapid growth rate.

Egg production takes place by reason of complex interactions of genes regulating energy metabolism, protein synthesis and storage processes, and this physiological phenomenon is dependent on a variety of organs involved in the reproduction^2^. The hypothalamic-pituitary-gonadal (HPG) axis regulates the reproductive and endocrine systems in laying hens^3^. In birds, the hypothalamus strictly regulates reproduction process by secreting gonadotropin-releasing hormone-I (GnRH-I) that stimulates the adenohypophysis (anterior pituitary gland) to synthesize and release Luteinizing hormone (LH) and Follicle Stimulating hormone (FSH). These gonadotrophins act on the gonads to stimulate gametogenesis and sex steroid hormone secretion from specialized cell in the left ovary. The ovary is extremely important for regulating oocyte development, follicle maturation and ovulation process by releasing of sex hormones^4^. In the ovary, the estrogen that mainly secreted by granulose cells is not only for local use but also transfers endocrine signals to other tissues^5^. However, both the capacity of the oviduct to transport the ova into a hard-shelled egg and the number of follicles prepared for ovulation determined the number of eggs laid by a chicken. Furthermore, beside environment and metabolism factors, the ovarian follicle growth and development is controlled by a mass of autocrine, paracrine and endocrine factors, including growth factors, sex steroid hormones and gonadotropins.

Recently, some studies have identified genes associated to reproduction traits at genomic and transcriptomic level^6^. For instance, Kang *et al*. (2009) identified twenty six differentially expressed genes in the ovaries between the pre-laying and egg-laying period^7^. Luan *et al*. (2014) identified twelve genes that are mainly involved in pathways for reproduction regulation, such as GnRH signaling pathways, steroid hormone biosynthesis, oocyte meiosis, progesterone-mediated oocyte maturation, G-protein coupled receptor signaling pathway, calcium signaling pathways and steroid biosynthesis^8^. In chicken, nine transcripts expressed specifically in the hypothalamus and pituitary gland have been identified that are associated to high egg production, providing a valuable resource as molecular markers of high egg production in poultry^9^. More recently, a transcriptome study on Chinese Dagu Chickens (CDC) reported putative differences in gene expression by comparing the hypothalamus and pituitary gland^10^. However, the chicken ovarian transcriptome has not yet been completely defined, and the major regulatory genes governing oogenesis and follicle development still remain unidentified^11^.

As the existing breeding programs are so time-consuming, inventing a fast and accurate method has become an important goal in exploring the most important genetic relationships. Recently, few RNA-sequencing attempts have been successfully used for chicken pituitary, hypothalamic and ovarian tissues to identify unique miRNA potentially associated to high and low egg-laying performance^12,13^. However, the precise molecular mechanism controlling the egg performance specifically on the HPO axis in chicken remains unclear. In order to uncover the unique genetic attributes responsible for egg production in Luhua chicken, the reproductive axis biology need to be explored.

The Luhua chickens (*Gallus gallus domesticus*) are native breed found in Wenshang county of Shandong province, China^14,15^. These are commercial dual-purpose egg and meat type chicken, famous for their superior reproductive performance such as high rates of egg production. In this study, we utilized high-throughput RNA sequencing technology to generate a comprehensive transcriptome profile of pituitary, ovary and hypothalamus tissues of Luhua chicken selected for high and low egg production at 300 days of age. The gene expression information at transcriptional level will aid understanding of the involvement of molecular factors related to egg production rate in Luhua chicken and hence can be used as selection criteria in chicken breeding process to improve egg production.

## Materials and methods

### Ethics statement

All methods were performed in accordance with the relevant guidelines and regulations provided by the Regulations of the Administration of Affairs Concerning Experimental Animals (China, 1998) for animal experiments. All experiments were reviewed and approved by the Committee on the Care and Use of Laboratory Animals of the State-level Animal Experimental Teaching Demonstration Center of Sichuan Agricultural University (Approval ID: S20141010). All the efforts were made to minimize the suffering of the chicken.

### Experimental animals and samples preparation

A total of 856 Chinese Luhua chicken cultivated in poultry breeding farm of Sichuan Agricultural University was chosen as objects in this study. All layers were housed in individual pen of the same feeding and management condition. Egg number of these chickens was recorded from age at first egg to 300 days of age. The birds were sorted according to the total egg number at 300 days of age (EN300) from high to low. The top 200 chickens with greater egg production were considered as high egg production (HEP) and the last 200 chickens with lesser egg production were considered as low egg production group (LEP) and Chickens with larger difference in body weight were excluded. We selected three chickens from each HEP and LEP group according to the same laying patterns as the final experimental birds. The reproductive traits such as body weight at first egg, body weight at 300 days of age, weight of first egg, egg weight at 300 days of age, age at first egg, and number of eggs at 300 days of age, were recorded for each chicken (Table S1). The average EN300 was 111 ± 2.1 and 144 ± 4.3 (Mean ± S.E.M.) for LEP and HEP chickens, respectively. Since then, chickens were sacrificed by humanitarian and hypothalamus, pituitary gland and ovary samples were collected (approximate 2 hr prior to ovulation) quickly. At the time of ovarian tissue collection, the egg yolk of the follicles was removed. All tissues were washed with 1X PBS, wrapped in zip locked polybags and frozen in liquid nitrogen, and then stored at −80 °C until RNA extraction.

### RNA extraction and quality control

Total RNA was extracted from all samples using RNeasy Mini Kit (Qiagen, Hilden, Netherlands) according to the manufacturer’s protocols. RNA purity was measured using the Nanodrop 2000 spectrophotometer and formaldehyde-agarose gel electrophoresis. RNA concentration and integrity were evaluated using the Qubit 2.0 Fluorometer (Thermo Fisher Scientific, Wilmington, DE) and the Agilent Bioanalyzer 2100 system (Agilent Technologies, CA, USA), respectively. A total 15 of the 18 RNA samples were selected for library preparation, while 3 sample of each tissue from same chicken was excluded due to poor quality.

### cDNA library construction and mRNA sequencing

The cDNA libraries were constructed with the NEBNext^®^ Ultra^TM^ RNA Library Prep Kit for Illumina^®^ (NEB, USA) following manufacturer’s protocols. Briefly, messenger RNA (mRNA) was isolated from total RNA using oligo(dT) coupled to magnetic beads, and short mRNA fragments (about 200 bp) were obtained through a fragmentation buffer. The first strand of cDNA was synthesized with random hexamer-primer using the mRNA fragments as template. Second strand of cDNA synthesis was then performed using Buffer, deoxynucleotide triphosphates (dNTPs), Ribonuclease H (RNase H) and DNA polymerase I. Subsequently, double-stranded cDNAs were purified with the QiaQuick PCR extraction kit (Qiagen, Germany) and eluted with EB buffer for end repair and poly(A) addition. Finally, sequencing adapters were ligated to the 5′ and 3′ ends of the fragments, the fragments were purified by agarose gel electrophoresis and enriched by PCR amplification to establish a cDNA library. The cDNA libraries were sequenced on the Illumina sequencing platform (HiSeq^TM^2500) at the Sangon Biotech Co., Ltd. in Shanghai, China.

### RNA-Seq data analysis

In order to ensure the quality of the samples, we used FastQC (v0.11.5) of raw reads with default parameters. The raw paired end reads were trimmed using the fastx_trimmer (fastx_toolkit-0.0.13.2) to remove low-quality reads, adaptor sequences, poly-N, >95% residual rates with clean mean length 95 bp was obtained from each sample. Clean reads were then mapped against the chicken reference genome *Gallus gallus* (v6.0) available in Ensembl v98 using HiSAT2.0 with default parameters. Reads number were enumerated and merged to a transcriptome using Stringtie^16^ as on resulting alignment and Ensembl annotation files.

### Identification of differentially expressed genes

The gene expression level was calculated based on its FPKM (Fragments per Kilobase of transcript per Million mapped reads) values using the Cufflinks package (v2.1.1). The expression pattern assessment for differentially expressed genes (DEGs) was performed in MultiExperiment Viewer (MeV, v4.9.0) software (https://sourceforge.net/projects/mev-tm4/files/mev-tm4/) based on normalized FPKM + 1 value between two groups. Genes with an adjusted P-value ≤0.05 were assigned as differentially expressed. P-values were calculated using *t*-test to judge the significance of the gene expression. Functional analysis for Gene Ontology (GO) terms and the KEEG pathways annotation were performed in Metascape (http://metascape.org) online server^17^.

### Hierarchical clustering and co-expression network analysis

Hierarchical cluster analysis of differentially expressed genes screened between pituitary, ovary and hypothalamus of high and low egg production chicken was performed in gplots package in R V.3.5.2 (http://www.r-project.org/). The co-expression network was constructed using Genemania^18^. The weighted network contemplates the significance of each gene in the input list. The network weights showed the aptitude of each gene.

## Results

### Sequencing and reads assembly

Through sequencing of seventeen cDNA libraries using the Illumina Hiseq. 2500 platform, a total 76.09 Gb RNA-seq raw sequences were obtained from the HPO axis (A: HEP and B: LEP) with an average of 5.07 Gb per sample and mean length of 126 bp. After stringent filtering, a total of 773,616,316 clean reads (HEP: 368,631,338 with an average of 46078917, LEP: 404,984,978 with an average of 44998330) were obtained. For HEP and LEP groups, an average of 38,123,661 and 38,644,495 reads were mapped to the reference genome with mapped rate of 84.07 and 85.52%, respectively with default parameters (Table S2).

### Analysis of differentially expressed genes

In order to explore the differentially expressed genes (DEGs) between HEP (A) & LEP (B) chickens for HPO axis in different tissues, gene expression levels were quantified by FPKM. A total of 414 DEGs were identified in the pituitary cDNA libraries, including 277 up-regulated genes and 137 down-regulated genes. In the ovary cDNA libraries, 356 DEGs were found, of which 214 were up-regulated and 142 were down-regulated. In the hypothalamic cDNA libraries, 10 DEGs were found, of them 8 were up-regulated and 2 were down-regulated (Table 1, Tables S3–S5). Venn-diagram analysis revealed that 8 DEGs displayed differential expression between pituitary and ovary tissues, and 1 DEG was identified in the comparison between pituitary and thalamus tissue (Fig. S1), in addition 405, 348 and 9 specifically detected in the pituitary, ovary, and thalamus groups, respectively, indicating different transcriptional changes in HPO axis during egg production.

**Table 1:** Statistics of DEGs in high egg production chicken, t-test, P ≤ 0.05.

Compared tissues	DEGs		
	up	down	All
B_pituitary gland vs A_pituitary gland	277	137	414
B_ovary vs A_ovary	214	142	356
B_hypothalamus vs A_hypothalamus	8	2	10

### Functional annotation and classification

**Figure 1:**
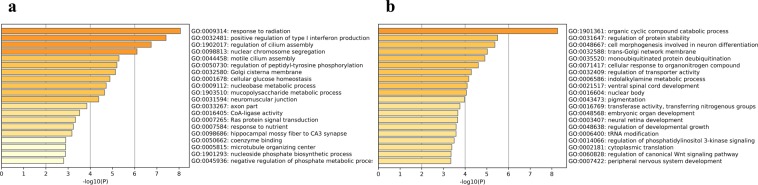
Gene Ontology (GO) analysis of differentially expressed genes between samples of HEP hens and LEP hens are shown by heatmap, using a discrete color scale to represent statistical significance. (**a**) Top 20 terms enriched in pituitary gland, (**b**) Top 20 terms enriched in ovary.

In comparison of pituitary gland between HEP and LEP group, the 414 DEGs were enriched to 108 different GO terms (including 79 biological process, 16 cellular components, 13 molecular functions), and top 20 were listed (P < 0.01, Fig. 1a, Table S6). The significant GO terms for biological processes were, GO:0009314 (response to radiation), GO:0032481 (positive regulation of type I interferon production), GO:1902017 (regulation of cilium assembly), GO:0098813 (nuclear chromosome segregation), GO:0044458 (motile cilium assembly), GO:0050730 (regulation of peptidyl-tyrosine), GO:0032580 (Golgi cisterna membrane), GO:0001678 (cellular glucose homeostasis), GO:0009112 (nucleobase metabolic process), and GO:1903510 (mucopolysaccharide metabolic process). Under cellular components GO terms were, GO:0031594 (neuromuscular junction), GO:0033267 (axon part), GO:0007265 (Ras protein signal transduction), and GO:0005815 (microtubule organizing center). The most relevant terms for molecular functions were, GO:0016405 (CoA-ligase activity), and GO:0050662 (coenzyme binding). The up-regulated 277 DEGs were enriched to 110 GO terms and the down-regulated 137 DEGs were enriched to 25 GO terms. In up-regulated DEGs, significant GO terms for biological processes were regulation of cilium assembly, nuclear chromosome segregation, motile cilium assembly, response to radiation, establishment of organelle localization, cellular glucose homeostasis, response to nutrient, positive regulation of kinase activity, mucopolysaccharide metabolic process, regulation of establishment of protein, and defense response to virus, and for molecular functions GO terms included protein kinase activity, and phosphotransferase activity, alcohol group as acceptor. In down-regulated DEGs, significant GO terms for biological processes were hindbrain development, forebrain development, mitochondrion organization.

In comparison of ovary between HEP and LEP group, the 356 DEGs were enriched to 93 different GO terms (including 83 biological process, 7 cellular components, 3 molecular functions), and top 20 were listed (P < 0.01, Fig. 1b, Table S7). The significant GO terms for biological processes were, GO:1901361 (organic cyclic compound catabolic process), GO:0048568 (embryonic organ development), GO:0060828 (regulation of canonical Wnt signaling), GO:0048667 (cell morphogenesis involved in neuron), GO:0043434 (response to peptide hormone), GO:0050953 (sensory perception of light stimulus), GO:0009792 (embryo development ending in birth or egg hatching). The most relevant term for molecular functions was, GO:0016769 (transferase activity, transferring nitrogenous). Under cellular components GO terms was, GO:0032588 (trans-Golgi network membrane) and GO:0032870 (cellular response to hormone stimulus). The up-regulated 214 DEGs were enriched to 80 GO terms and the down-regulated 142 DEGs were enriched to 46 GO terms. In up-regulated DEGs, significant GO terms for biological processes were regulation of glutamate receptor signaling pathway, phosphatidylinositol 3-kinase signaling, positive regulation of nervous system, anterograde trans-synaptic signaling, and for cellular components GO terms included trans-Golgi network membrane, nuclear body, and nuclear speck. In down-regulated DEGs, significant GO terms for biological processes were response to lipid catabolic process phospholipid metabolic process, embryonic organ development, and response to extracellular stimulus. Due to the smaller number of DEGs (10) identified in thalamus tissue between HEP versus LEP chicken group, the GO term could not be assigned.

### Pathway analysis

To further define the contribution of specific signaling pathways in egg production, KEGG pathway/enrichment analysis was performed representing the differentially expressed genes in HEP and LEP chickens. In pituitary gland, 414 DEGs were significantly enriched in 4 KEGG pathways (Table 2). In detail, the mTOR signaling pathway and Jak-STAT signaling pathway were the prominent KEGG pathway enriched in pituitary gland (p < 0.05). Genes corresponding to mTOR signaling pathway included *CAB39L* and *LPIN1* (upregulated) and *SOS1, FZD7, LAMTOR1, CAB39L, WDR24* (downregulated). The Jak-STAT signaling pathway consisted of *IL2RA*, *IL13RA1*, *IL15* (upregulated) and *CREBBP*, *IL12RB2*, *SOS1*, *SOCS4* (downregulated).

**Table 2:** Lists of the significantly enriched KEGG pathways associated with Pituitary gland and ovary.

Tissue	Pathways	Term	DEGs No.	Log10(P)	Genes	
					Up-regulated	Down-regulated
Pituitary gland	Long-term potentiation	gga04720	4	−9.56915	CALML3	CREBBP, GRIN1, RPS6KA1,
	Butanoate metabolism	gga00650	4	−5.44359	GAD2, HADH, HMGCS1, AACS	
	mTOR signaling pathway	gga04150	7	−2.93171	LPIN1, CAB39L,	RPS6KA1, SOS1, FZD7, LAMTOR1, WDR24
	Jak-STAT signaling pathway	gga04630	7	−2.85	IL2RA, IL13RA1, IL15	IL12RB2, SOS1, SOCS4
Ovary	mRNA surveillance pathway	gga03015	3	−6.28867	CSTF1, PPP2R2A, PPP2R5C	
	Ribosome	gga03010	5	−3.70537		RPL7, RPL12, RPL30, RPL27A, RPL34
	Tryptophan metabolism	gga00380	3	−3.43741	KYNU, TDO2	AANAT
	PI3K-Akt signaling pathway	gga04151	7	−2.19303	FGF14, PPP2R2A, PPP2R5C, RELN, RBL2,	FGF23, LAMB4

In ovary, 356 DEGs were significantly enriched in 4 KEGG pathways, including Tryptophan metabolism (*TDO2, KYNU*), and PI3K-Akt signaling pathway (*GH1*, *PPP2R2A*, *PPP2R5C*, *RELN*, *RBL2*, *FGF23*, *LAMB4*) were related to reproductive regulation (Table 2). However, no significant enrichment of the KEGG pathway was found in the thalamus of HEP versus LEP chickens.

### Hierarchical clustering analysis of differentially expressed genes

**Figure 2:**
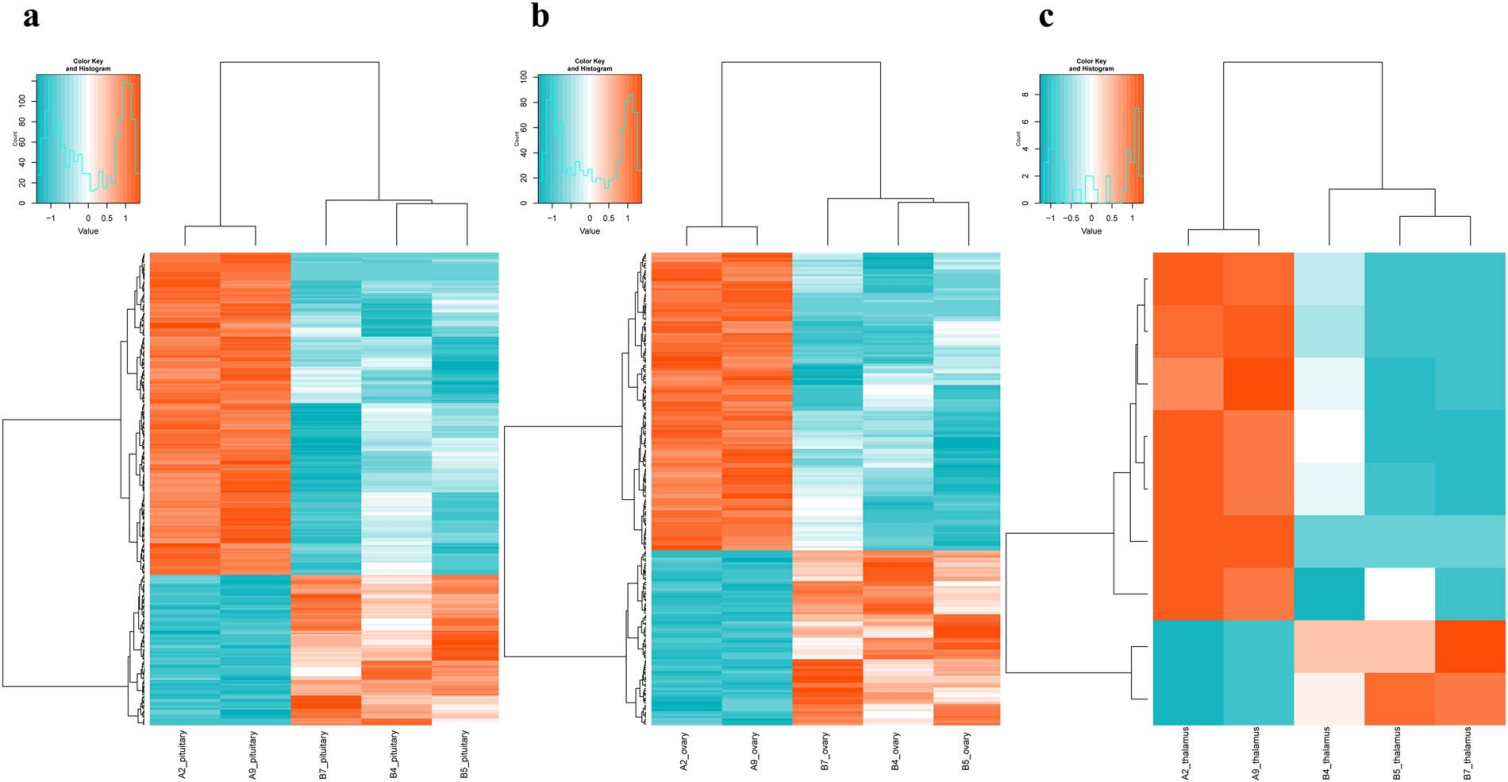
Hierarchical clustering analysis of the significantly DEGs down- or up-regulated between samples of HEP hens and LEP hens. Heatmap displaying (**a)** 414 DEGs in pituitary gland (**b)** 356 DEGs in ovary (**c)** 10 DEGs in ovary. (red, upregulated; green, downregulated).

Hierarchical clustering analysis was carried out based on FPKM visually reflects expression profile of significant DEGs screened between the pituitary, ovary and hypothalamus tissues of HEP and LEP chicken groups (Fig. 2a–c). The rows represent genes differentially expressed, and columns represent different samples, the red color indicates high expression level and green indicates to low expression level. Hierarchical cluster above the map shows clustering of tissues and cluster of left map shows that similar expression patterns were clustered.

### Interaction between DE genes

**Figure 3:**
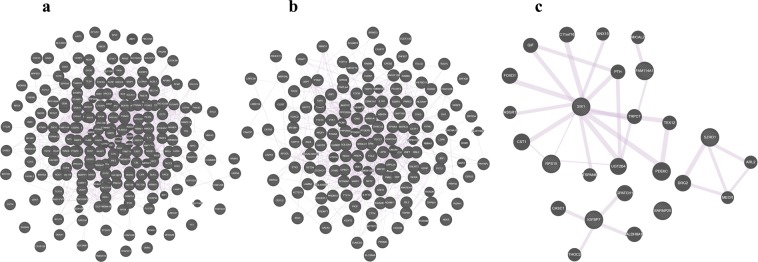
The co-expression network of significantly DEGs genes between samples of HEP hens and LEP hens. (**a)** pituitary gland, (**b)** ovary, (**c)** hypothalamus.

A co-expression network was constructed between 277 upregulated DEGs appeared in pituitary of HEP group, p < 0.05 (Fig. 3a). A total of 1302 interactions were observed in the DEGs of pituitary gland. The most relevant genes based on topmost network weights consists of *ACSL1, HMGCR, HMGCS1, NFKB1, VAV3* genes, which are involve in regulation of regulation of lipid metabolic process. These genes were found to be up-regulated in the pituitary gland of HEP chickens. Moreover, few more highly connected DEGs such as *IRF1*, *NFKB1*, *SOS1* were found to be associated with Prolactin signaling pathway.

In ovary, a total of 602 interactions were observed in the 214 DEGs (Fig. 3b). The most relevant genes based on topmost network weights consists of *IL1R1*, *MEF2C*, *STK3* genes, which are involve in regulation of MAPK signaling pathway. These genes were found to be up-regulated in the ovary of HEP chickens. Moreover, *TGFBR2* (embryo development ending in birth or egg hatching and *AKIRIN2* (in utero embryonic development) were also found to be upregulated in HEP group.

In hypothalamus, a total of 30 interactions were observed in the 10 DEGs (Fig. 3c). The most relevant genes based on topmost network weights included *SIX1*, *RPS15*, *IGFBP7* genes.

## Discussion

Domestic chickens are one of the major agricultural poultry species worldwide and has a significant economic value by providing meet and egg. In birds, hormones of HPO axis are involved in the whole reproductive regulation. In this process, the hypothalamus produces gonadotropin-releasing hormone-I (GnRH-I) which triggers pituitary gland (adenohypophysis) to release FSH and LH, and stimulates the secretion of estradiol (E2) and progesterone (P4) in ovary while ovulation^19^. Hitherto, several studies have focused to uncover the molecular mechanism associated with egg production performance in chicken^10,20,21^. However, the differential gene expression and key pathways mediating the egg production process across hypothalamic-pituitary-ovarian (HPO) axis are yet to be uncovered. In the present study, whole transcriptome of the hypothalamic-pituitary-gonadal axis of chicken was used to understand the regulatory mechanism of DEGs involve in high and low egg production chicken.

In this study, a deep mRNA expression profile was generated for the pituitary gland, ovary and hypothalamus from HEP and LEP Luhua chickens. To best of our knowledge, this is the first report to provide comprehensive view of the mRNA level changes exhibited across HPO axis in chickens of relatively high and low rate of egg production using RNA-seq technology. In total, 414, 356, and 10 DEGs were identified using MeV (P < 0.05, t-test) in the pituitary gland, ovary and hypothalamus between HEP and LEP chickens, respectively.

DEGs in the pituitary gland were mainly enriched in response to response to radiation, brain development, lipoprotein biosynthetic process, cartilage development, negative regulation of hormone secretion. Several DEGs such as *HADH*, *HMGCR*, *RAB11FIP1*, *FAM3D* are found to be involved in hormonal regulation related to reproduction performance. Research shows that three variants (G-789-A, C-937-G, and A-2316-C) of 3-Hydroxy-3-methylglutaryl-CoA reductase (*HMGCR*) gene are associated with chicken egg production and very low-density lipoprotein (VLDL) concentrations showed lower egg production^22^. Apoplipoprotein B (*APOB*) is primary organizing protein of chylomicrons and VLDL, and is responsible for lipoprotein transport^23,24^. In chicken, liver lipoproteins are circulated in the plasma and stored into the oocytes to form the egg yolk in laying birds. KEGG pathway analysis showed that the DEGs identified in pituitary gland between HEP and LEP chickens were mainly involved in signaling pathways such as mTOR signaling pathway and Jak-STAT signaling pathway. The mammalian target of Rapamycin (mTOR) signaling pathway recently been examined in ovarian follicles in mouse where it regulates granulosa cell proliferation and differentiation^25^.

The GO terms in ovary showed that DEGs were enriched in embryonic organ development, glial cell migration, regulation of canonical Wnt signaling pathway, peripheral nervous system development embryo development ending in birth or egg hatching. Several DEGs such as *GDNF, HOXD9, MEF2C, STK3, CLRN1, IRX5, LBX1* involved in embryonic organ development and genes *CSNK1A1, STK3, LGR5, PRDM15, DAB2IP* were found to be up-regulated in high egg production chicken. DEGs in ovary mainly enriched to 4 KEGG pathways, including Tryptophan metabolism PI3K-Akt signaling pathway were important for the egg production. Previous studies revealed that stressful situations can alter peripheral and brain tryptophan levels by stimulating the immune system and activating the hypothalamic-pituitary-adrenal axis^26,27^. The tryptophan-2,3-dioxygenase (*TDO*) gene mainly expressed in brain and is activated by hormones (glucocorticoids, prolactin)^28^.

In hypothalamus, expression level of *EXFABP, SNRNP25, FAM114A1, SIX1* genes was increased in high rate of egg production hens compared with low rate of egg production hens. Previously, an investigation on the effects of dietary corticosterone reported that after treatment extracellular fatty acid-binding protein (EXFABP) was decreased, suggesting environmental stress play crucial role in synthesis and secretion of egg white proteins in laying hens^29^.

In order to understand the relationship between differentially expressed genes and its transcription factors, it is important to analyze their connectivity with other molecules or regulators^18^. Network analysis of DEGs in pituitary gland showed co-expression of genes such as *HMGCR, HMGCS1, NFKB1, VAV3, SOS1, IL1R1, MEF2C, STK3* were highly connected. These genes were mainly involved in regulation of lipid metabolic process, prolactin signaling pathway, MAPK signaling pathway. Previous studies revealed that prolactin (PRL) is synthesized and released from the anterior pituitary gland and involves in several physiological process such as reproduction behavior, egg-laying, metabolism, development and regulation of hypothalamic–pituitary–gonadal axis in chicken^30,31^. Highly connected genes in ovary involved in regulation of protein catabolic process, embryo development. NF-kappaB-dependent gene expression of *AKIRIN2* nuclear protein has been reported in the ovary of mouse and other rodents^32^. In hypothalamus, *SIX1, RPS15, IGFBP7* genes were found to be highly connected. These genes were found to be function in response to nerve growth factor, lipid metabolism, canonical Wnt signaling pathway, thus play vital role in egg reproduction process in chicken. Numerous studies have reported that insulin-like growth factor binding proteins (IGFBPs) are stimulators of ovarian follicular development, and play crucial role in in the responsiveness of the ovary to FSH action^33,34^. A previous study revealed that insulin-like growth factor binding protein 7 (IGFBP7) involved in lipid metabolism was highly expressed in adipose tissue of chickens^35^. In a previous study, it has been shown that there is a correlation between *IGF-1* gene expression and egg production traits in laying chickens^36^.

## Conclusion

In this study, we identified key differentially expressed genes involved in regulation of pathways associated with reproductive functions in the chicken hypothalamic-pituitary-ovarian axis using RNA-sequencing. Findings in our research could better understand the transcriptional basis of high and low rate of egg production in Luhua chicken. The potential genes identified in this study can be used as selection marker in Luhua chicken to increase laying performance, however, functional validation experiment is needed in other poultry species.

## Supplementary information

Table S1-S7 and Figure S1.

## Data Availability

The sequencing data for the HPO axis transcriptome through Illumina HiSeq. 2500 have been deposited in the National Center for Biotechnology Information (NCBI) Sequence Read Archive under the accession number SRP234990.
