# Supplementary material for: Transcriptome analysis reveals differentially expressed genes associated with high rates of egg production in chicken hypothalamic-pituitary-ovarian axis
Source: Sci Rep. 2020 Apr 6;10:5976. doi: 10.1038/s41598-020-62886-z (PMC7136225; doi:10.1038/s41598-020-62886-z)
Supplement: Supplementary file 1 — Table S1-S7 and Figure S1. [file 41598_2020_62886_MOESM1_ESM.docx]

**Transcriptome analysis reveals differentially expressed genes associated with high rates of egg production in chicken hypothalamic-pituitary-ovarian axis**

Shailendra Kumar Mishra^1^, Binlong Chen^2^, Qing Zhu^1^*, Zhongxian Xu^1^, Chunyou Ning^1^, Huadong Yin^1^, Yan Wang^1^, Xiaoling Zhao^1^, Xiaolan Fan^1^, Mingyao Yang^1^, Deying Yang^1^, Qingyong Ni^1^, Yan Li^1^, Mingwang Zhang^1^ and Diyan Li^1^*

^1^Farm Animal Genetic Resources Exploration and Innovation Key Laboratory of Sichuan Province, Sichuan Agricultural University, Chengdu, 611130, China; ^2^XiChang University, Xuefu road No 1, Anning town, Xichang, Liangshan prefecture, Sichuan province, China.

Shailendra Kumar Mishra, Binlong Chen and Qing Zhu contribute equally to this work.

**Corresponding author: Qing Zhu and Diyan Li**

^1^Farm Animal Genetic Resources Exploration and Innovation Key Laboratory of Sichuan Province, Sichuan Agricultural University, Chengdu, 611130, Chin

E-mail: [zhuqingsicau@163.com](mailto:zhuqingsicau@163.com) and [diyanli@sicau.edu.cn](mailto:diyanli@sicau.edu.cn)

**Table S1. Reproductive traits recorded during laying period**

| **Chicken** | **AFE** | **BWFE** | **WFE** | **EW300** | **BW300** | **EN300** |
| --- | --- | --- | --- | --- | --- | --- |
| A2 | 156 | 1.4 | 36.2 | 51.5 | 1.9 | 140 |
| A3 | 155 | 1.5 | 37.1 | 49 | 1.9 | 150 |
| A9 | 153 | 1.42 | 35.7 | 52.1 | 2.1 | 142 |
| B4 | 157 | 1.53 | 35.3 | 48.4 | 2.1 | 112 |
| B5 | 151 | 1.4 | 37.4 | 52.7 | 2.1 | 108 |
| B7 | 156 | 1.51 | 38.2 | 53.8 | 2.1 | 113 |

High egg production (HEP): A2, A3, A9; low egg production (LEP): B4, B5, B7; Age= age at first egg; BWFE = body weight at 1st egg; WFE (g)= weight of first egg; EW300 = egg weight at 300 days; BW300 = body weight at 300 days of age; EN300 = egg number at 300 days.

**Table S2.** Reads Mapping to the reference genome dataset

| **Sample** | **Raw reads** | **Clean reads** | **Mapped Reads** | **Mapped Ratio** | **Q20(%)** |
| --- | --- | --- | --- | --- | --- |
| A2_pituitary gland | 73965180 | 64785370 | 54643007 | 84.34 | 91.55 |
| A2_ovary | 44134624 | 40865754 | 38091161 | 93.21 | 94.81 |
| A2_hypothalamus | 44311306 | 38352290 | 30559312 | 79.68 | 91.18 |
| A9_pituitary gland | 41014294 | 37121140 | 29694660 | 79.99 | 93.02 |
| A9_ovary | 45775774 | 42270116 | 39051333 | 92.39 | 94.44 |
| A9_hypothalamus | 56298810 | 49062622 | 36702496 | 74.81 | 91.32 |
| B4_pituitary gland | 46107884 | 39916898 | 33510245 | 83.95 | 90.93 |
| B4_ovary | 61087810 | 55501044 | 54030763 | 97.35 | 93.23 |
| B4_hypothalamus | 61789226 | 54253294 | 39844160 | 73.44 | 91.70 |
| B5_pituitary gland | 53321432 | 45666278 | 40043426 | 87.69 | 90.49 |
| B5_ovary | 61533958 | 55808254 | 51395255 | 92.09 | 93.05 |
| B5_hypothalamus | 39279732 | 34285464 | 27406777 | 79.94 | 91.59 |
| B7_pituitary gland | 48398388 | 43851182 | 35414100 | 80.76 | 92.99 |
| B7_ovary | 42205502 | 38951658 | 36392852 | 93.43 | 94.37 |
| B7_hypothalamus | 41752324 | 36750906 | 29762877 | 80.99 | 91.92 |

**Table S3.** List of DEGs in pituitary gland samples between HEP and LEP chickens

| **GeneID** | **Mean-HEP** | **Mean-LEP** | **Adj p value** | **log2Ratio (LEP/HEP)** | **Type** |  |
| --- | --- | --- | --- | --- | --- | --- |
| ENSGALG00000047191 | 2.73142 | 1 | 0.011555865 | 1.449651169 | Up |  |
| ENSGALG00000017005 | 45.304367 | 19.491648 | 0.013390043 | 1.216794048 | Up |  |
| ENSGALG00000043671 | 9.612113 | 4.3142886 | 0.04151744 | 1.155730922 | Up |  |
| ENSGALG00000019941 | 6.586233 | 3.1048877 | 0.028742181 | 1.084912462 | Up |  |
| ENSGALG00000028063 | 5.9783754 | 2.8485124 | 0.049177706 | 1.069544804 | Up |  |
| ENSGALG00000009017 | 10.83781 | 5.5131016 | 0.042006012 | 0.975137166 | Up |  |
| ENSGALG00000032506 | 12.153046 | 6.460974 | 0.04450968 | 0.911494377 | Up |  |
| ENSGALG00000016456 | 13.387901 | 7.1464653 | 0.046159904 | 0.905628033 | Up |  |
| ENSGALG00000011566 | 5.1274023 | 2.7718961 | 0.022711236 | 0.887354916 | Up |  |
| ENSGALG00000013948 | 3.2238765 | 1.7936239 | 0.026218418 | 0.845919071 | Up |  |
| ENSGALG00000027258 | 26.855785 | 15.010757 | 0.015103456 | 0.839236158 | Up |  |
| ENSGALG00000051690 | 1.814517 | 1.0496136 | 0.006298355 | 0.789727255 | Up |  |
| ENSGALG00000039978 | 3.050854 | 1.8263794 | 0.008821476 | 0.740226648 | Up |  |
| ENSGALG00000042471 | 1.9196429 | 1.1548067 | 0.019196406 | 0.733186577 | Up |  |
| ENSGALG00000052466 | 13.067119 | 7.9566216 | 0.02737188 | 0.715713201 | Up |  |
| ENSGALG00000017126 | 19.487623 | 11.960185 | 0.041402098 | 0.704318424 | Up |  |
| ENSGALG00000039965 | 4.117223 | 2.5337427 | 0.044611026 | 0.700401564 | Up |  |
| ENSGALG00000012704 | 79.92426 | 49.32489 | 0.04082218 | 0.696317648 | Up |  |
| ENSGALG00000048789 | 2.9629514 | 1.8502097 | 0.018551925 | 0.67934617 | Up |  |
| ENSGALG00000029565 | 17.34256 | 10.829829 | 0.019907739 | 0.679306413 | Up |  |
| ENSGALG00000020210 | 2.78052 | 1.7629074 | 0.033147126 | 0.657398018 | Up |  |
| ENSGALG00000015590 | 8.251406 | 5.277311 | 0.034109686 | 0.644836962 | Up |  |
| ENSGALG00000009468 | 3.650592 | 2.3415098 | 0.026989317 | 0.640691362 | Up |  |
| ENSGALG00000013311 | 4.093808 | 2.631506 | 0.03019324 | 0.637554757 | Up |  |
| ENSGALG00000043386 | 5.0510345 | 3.260683 | 0.024493592 | 0.631404705 | Up |  |
| ENSGALG00000052533 | 2.2086954 | 1.4493927 | 0.047697775 | 0.607745938 | Up |  |
| ENSGALG00000013583 | 6.681329 | 4.4023166 | 0.023124872 | 0.601872199 | Up |  |
| ENSGALG00000017136 | 13.74118 | 9.08937 | 0.041273613 | 0.596253691 | Up |  |
| ENSGALG00000054834 | 1.6730316 | 1.1135901 | 0.014682367 | 0.587246405 | Up |  |
| ENSGALG00000028222 | 2.3063173 | 1.5456208 | 0.028406259 | 0.577404596 | Up |  |
| ENSGALG00000021405 | 26.56863 | 17.859056 | 0.015019608 | 0.573068014 | Up |  |
| ENSGALG00000035447 | 9.6741085 | 6.5066543 | 0.03703888 | 0.572212813 | Up |  |
| ENSGALG00000004379 | 9.089342 | 6.11948 | 0.046836272 | 0.570766792 | Up |  |
| ENSGALG00000007152 | 1.6844475 | 1.139723 | 0.026143746 | 0.563592232 | Up |  |
| ENSGALG00000026907 | 2.4325776 | 1.648469 | 0.033095524 | 0.561359071 | Up |  |
| ENSGALG00000013207 | 8.209694 | 5.578633 | 0.04104128 | 0.557416805 | Up |  |
| ENSGALG00000009870 | 14.328789 | 9.760114 | 0.03257161 | 0.553946781 | Up |  |
| ENSGALG00000002899 | 34.058308 | 23.25077 | 0.0449427 | 0.550728269 | Up |  |
| ENSGALG00000040475 | 7.5034895 | 5.1855206 | 0.030596629 | 0.533072842 | Up |  |
| ENSGALG00000028274 | 1.7947226 | 1.2450147 | 0.046507202 | 0.527598096 | Up |  |
| ENSGALG00000001167 | 1.5211694 | 1.0636407 | 0.020854099 | 0.516169935 | Up |  |
| ENSGALG00000049912 | 2.177566 | 1.5263094 | 0.03228357 | 0.512669004 | Up |  |
| ENSGALG00000047218 | 1.419061 | 1 | 0.009017069 | 0.504936607 | Up |  |
| ENSGALG00000011549 | 4.773575 | 3.3648188 | 0.013821645 | 0.504541311 | Up |  |
| ENSGALG00000008778 | 17.956654 | 12.704728 | 0.04824341 | 0.499153058 | Up |  |
| ENSGALG00000039327 | 8.906923 | 6.3187637 | 0.028328221 | 0.495284807 | Up |  |
| ENSGALG00000010246 | 19.33221 | 13.725553 | 0.049970776 | 0.494142295 | Up |  |
| ENSGALG00000007956 | 13.447056 | 9.569793 | 0.03182216 | 0.49073073 | Up |  |
| ENSGALG00000016364 | 3.8141885 | 2.719721 | 0.031559408 | 0.487917483 | Up |  |
| ENSGALG00000010502 | 6.939019 | 4.9520845 | 0.04567277 | 0.486695785 | Up |  |
| ENSGALG00000042621 | 2.2609355 | 1.6261287 | 0.017380718 | 0.475478391 | Up |  |
| ENSGALG00000044418 | 1.5542569 | 1.1179957 | 0.031735726 | 0.475310344 | Up |  |
| ENSGALG00000044480 | 15.040055 | 10.82262 | 0.049764328 | 0.474760046 | Up |  |
| ENSGALG00000035799 | 10.75375 | 7.7684684 | 0.03504268 | 0.469137742 | Up |  |
| ENSGALG00000013603 | 6.636901 | 4.8179545 | 0.046187285 | 0.462088985 | Up |  |
| ENSGALG00000016922 | 13.832869 | 10.07213 | 0.04571524 | 0.4577316 | Up |  |
| ENSGALG00000015709 | 17.550766 | 12.787327 | 0.04936881 | 0.456819276 | Up |  |
| ENSGALG00000006421 | 20.11013 | 14.652629 | 0.034225594 | 0.456762869 | Up |  |
| ENSGALG00000015593 | 11.801166 | 8.615869 | 0.044623017 | 0.453861191 | Up |  |
| ENSGALG00000038231 | 4.37571 | 3.2049198 | 0.013749448 | 0.449228871 | Up |  |
| ENSGALG00000049090 | 2.078668 | 1.5243281 | 0.014790124 | 0.447485887 | Up |  |
| ENSGALG00000014862 | 57.910217 | 42.52285 | 0.027720014 | 0.445579609 | Up |  |
| ENSGALG00000005552 | 13.199301 | 9.703644 | 0.048903063 | 0.443863002 | Up |  |
| ENSGALG00000001618 | 9.531889 | 7.0508475 | 0.024287587 | 0.434965474 | Up |  |
| ENSGALG00000007030 | 5.3608375 | 3.9748676 | 0.009596961 | 0.431551598 | Up |  |
| ENSGALG00000009686 | 4.004 | 2.9758065 | 0.030251643 | 0.428161255 | Up |  |
| ENSGALG00000014186 | 14.364109 | 10.696488 | 0.011739721 | 0.425331315 | Up |  |
| ENSGALG00000000818 | 5.265025 | 3.9232147 | 0.049329627 | 0.424404092 | Up |  |
| ENSGALG00000003353 | 6.000181 | 4.4751983 | 0.047765046 | 0.42305441 | Up |  |
| ENSGALG00000011227 | 13.417878 | 10.028981 | 0.018276146 | 0.419981504 | Up |  |
| ENSGALG00000012100 | 2.4248228 | 1.813897 | 0.0470451 | 0.418786786 | Up |  |
| ENSGALG00000016897 | 13.242172 | 9.91269 | 0.038629524 | 0.417791256 | Up |  |
| ENSGALG00000054801 | 1.335125 | 1 | 0.036707945 | 0.416974819 | Up |  |
| ENSGALG00000052583 | 1.3327556 | 1 | 0.027480086 | 0.414412244 | Up |  |
| ENSGALG00000011046 | 26.443972 | 19.885735 | 0.016711239 | 0.411205005 | Up |  |
| ENSGALG00000009029 | 6.5838284 | 4.9525495 | 0.04234087 | 0.410755339 | Up |  |
| ENSGALG00000016124 | 72.87042 | 54.96135 | 0.04482851 | 0.406915865 | Up |  |
| ENSGALG00000041296 | 12.80553 | 9.683335 | 0.00444131 | 0.403191054 | Up |  |
| ENSGALG00000009807 | 9.203374 | 6.969715 | 0.026383644 | 0.401063193 | Up |  |
| ENSGALG00000006378 | 51.010803 | 38.635452 | 0.04600192 | 0.400877535 | Up |  |
| ENSGALG00000021773 | 1.319826 | 1 | 0.014120376 | 0.400347744 | Up |  |
| ENSGALG00000015349 | 11.199881 | 8.48717 | 0.03813768 | 0.400127923 | Up |  |
| ENSGALG00000042116 | 1.7316215 | 1.3125733 | 0.019426998 | 0.399725627 | Up |  |
| ENSGALG00000036582 | 4.007217 | 3.0417259 | 0.031022057 | 0.397710484 | Up |  |
| ENSGALG00000004425 | 13.236076 | 10.089565 | 0.048098806 | 0.391611505 | Up |  |
| ENSGALG00000054646 | 2.9394035 | 2.2408726 | 0.020630311 | 0.391462785 | Up |  |
| ENSGALG00000001561 | 8.508646 | 6.493153 | 0.03779585 | 0.390010366 | Up |  |
| ENSGALG00000016811 | 23.235527 | 17.74761 | 0.04666529 | 0.388707611 | Up |  |
| ENSGALG00000008424 | 1.3914545 | 1.0635604 | 0.03836754 | 0.387691767 | Up |  |
| ENSGALG00000034216 | 35.283432 | 26.973215 | 0.016828705 | 0.387463408 | Up |  |
| ENSGALG00000012903 | 7.7461452 | 5.9300427 | 0.003579872 | 0.385436052 | Up |  |
| ENSGALG00000016498 | 1.3440275 | 1.0298637 | 0.020242082 | 0.384109244 | Up |  |
| ENSGALG00000016675 | 7.573682 | 5.8131604 | 0.010212655 | 0.381672131 | Up |  |
| ENSGALG00000009413 | 14.48807 | 11.130253 | 0.047178432 | 0.380379035 | Up |  |
| ENSGALG00000027454 | 2.6747274 | 2.059358 | 0.047851313 | 0.377197213 | Up |  |
| ENSGALG00000009560 | 94.99367 | 73.18668 | 0.04353134 | 0.37625028 | Up |  |
| ENSGALG00000033068 | 6.081314 | 4.687614 | 0.04560335 | 0.375529307 | Up |  |
| ENSGALG00000015665 | 3.0505564 | 2.3529823 | 0.03382288 | 0.374581936 | Up |  |
| ENSGALG00000035935 | 2.313816 | 1.7856493 | 0.00492261 | 0.373825378 | Up |  |
| ENSGALG00000017139 | 10.259015 | 7.917484 | 0.021806192 | 0.373778268 | Up |  |
| ENSGALG00000038612 | 6.8667707 | 5.303102 | 0.022254432 | 0.372795291 | Up |  |
| ENSGALG00000045388 | 3.0915074 | 2.393964 | 0.037634145 | 0.368909001 | Up |  |
| ENSGALG00000020895 | 1.923092 | 1.4914913 | 0.036075797 | 0.36667222 | Up |  |
| ENSGALG00000014948 | 40.336395 | 31.292913 | 0.041689944 | 0.366246189 | Up |  |
| ENSGALG00000035785 | 4.494586 | 3.488722 | 0.04009592 | 0.365489594 | Up |  |
| ENSGALG00000009157 | 1.441999 | 1.1195676 | 0.018303348 | 0.365128523 | Up |  |
| ENSGALG00000011389 | 2.4256248 | 1.885365 | 0.01789627 | 0.363512558 | Up |  |
| ENSGALG00000023437 | 11.288172 | 8.774285 | 0.034418367 | 0.363458403 | Up |  |
| ENSGALG00000008745 | 10.107271 | 7.856527 | 0.045505937 | 0.363429906 | Up |  |
| ENSGALG00000015029 | 8.958312 | 6.970075 | 0.010118256 | 0.362052733 | Up |  |
| ENSGALG00000001577 | 29.038727 | 22.606895 | 0.048100494 | 0.361215355 | Up |  |
| ENSGALG00000010628 | 14.771657 | 11.505834 | 0.045981806 | 0.360466107 | Up |  |
| ENSGALG00000002044 | 21.790249 | 17.004658 | 0.036175888 | 0.357752692 | Up |  |
| ENSGALG00000026276 | 2.053642 | 1.6080984 | 0.0428261 | 0.352829018 | Up |  |
| ENSGALG00000014682 | 19.83787 | 15.5555315 | 0.019196425 | 0.350829442 | Up |  |
| ENSGALG00000038635 | 70.56305 | 55.412617 | 0.045681357 | 0.348698417 | Up |  |
| ENSGALG00000009112 | 11.531892 | 9.065955 | 0.023753837 | 0.347098325 | Up |  |
| ENSGALG00000006258 | 40.263466 | 31.673065 | 0.017637782 | 0.346214886 | Up |  |
| ENSGALG00000029673 | 12.636045 | 9.94352 | 0.024744235 | 0.34571642 | Up |  |
| ENSGALG00000037940 | 12.194756 | 9.621454 | 0.014236925 | 0.341934056 | Up |  |
| ENSGALG00000004294 | 8.6091 | 6.7984123 | 0.04991142 | 0.340664566 | Up |  |
| ENSGALG00000001988 | 6.6975574 | 5.3384247 | 0.036614027 | 0.327220956 | Up |  |
| ENSGALG00000003862 | 15.732771 | 12.5581665 | 0.023610165 | 0.325146949 | Up |  |
| ENSGALG00000019553 | 1.6083186 | 1.2857736 | 0.027538119 | 0.322916591 | Up |  |
| ENSGALG00000005643 | 2.590763 | 2.0711975 | 0.02119438 | 0.322911917 | Up |  |
| ENSGALG00000029432 | 1.6136434 | 1.2926193 | 0.007091459 | 0.320024354 | Up |  |
| ENSGALG00000006153 | 24.467693 | 19.601618 | 0.03432155 | 0.319905334 | Up |  |
| ENSGALG00000010680 | 12.32365 | 9.918072 | 0.003079391 | 0.313298011 | Up |  |
| ENSGALG00000015705 | 6.4958706 | 5.2320848 | 0.015950877 | 0.31213697 | Up |  |
| ENSGALG00000053281 | 10.512507 | 8.469089 | 0.049245875 | 0.311828065 | Up |  |
| ENSGALG00000002067 | 11.663704 | 9.399817 | 0.027220137 | 0.311321438 | Up |  |
| ENSGALG00000004247 | 3.7981381 | 3.0614412 | 0.033896424 | 0.311081389 | Up |  |
| ENSGALG00000002800 | 2.3827965 | 1.9227028 | 0.04411985 | 0.309519968 | Up |  |
| ENSGALG00000029582 | 25.391407 | 20.504446 | 0.023238862 | 0.308403576 | Up |  |
| ENSGALG00000032558 | 1.448143 | 1.1694278 | 0.004635809 | 0.308401278 | Up |  |
| ENSGALG00000017104 | 16.272537 | 13.147488 | 0.025849955 | 0.307652015 | Up |  |
| ENSGALG00000003695 | 8.537839 | 6.9005356 | 0.04890692 | 0.307162613 | Up |  |
| ENSGALG00000003129 | 11.436636 | 9.260437 | 0.04835408 | 0.304510576 | Up |  |
| ENSGALG00000039005 | 1.233746 | 1 | 0.036140878 | 0.303045407 | Up |  |
| ENSGALG00000014831 | 8.022357 | 6.502741 | 0.036184005 | 0.302978205 | Up |  |
| ENSGALG00000016522 | 5.365446 | 4.349375 | 0.035278328 | 0.302889997 | Up |  |
| ENSGALG00000052885 | 3.0394905 | 2.4668753 | 0.030789651 | 0.30114472 | Up |  |
| ENSGALG00000015334 | 8.799563 | 7.1466985 | 0.040882602 | 0.300154953 | Up |  |
| ENSGALG00000042356 | 3.6410975 | 2.9642603 | 0.022370627 | 0.296701233 | Up |  |
| ENSGALG00000014916 | 30.887135 | 25.154305 | 0.04694103 | 0.296200728 | Up |  |
| ENSGALG00000005815 | 18.163803 | 14.807786 | 0.03956572 | 0.294710344 | Up |  |
| ENSGALG00000001270 | 14.031862 | 11.451901 | 0.03794101 | 0.293119361 | Up |  |
| ENSGALG00000042338 | 2.606913 | 2.1320417 | 0.019407265 | 0.290106781 | Up |  |
| ENSGALG00000052407 | 2.0046282 | 1.6424732 | 0.014830225 | 0.287464853 | Up |  |
| ENSGALG00000006354 | 1.338552 | 1.0967709 | 0.017242502 | 0.287410987 | Up |  |
| ENSGALG00000008739 | 10.733749 | 8.798148 | 0.048561417 | 0.286882282 | Up |  |
| ENSGALG00000037177 | 14.262623 | 11.69255 | 0.04154775 | 0.28664973 | Up |  |
| ENSGALG00000029077 | 10.048305 | 8.245687 | 0.022103263 | 0.285240557 | Up |  |
| ENSGALG00000002854 | 3.332535 | 2.749855 | 0.045948196 | 0.27726448 | Up |  |
| ENSGALG00000014413 | 9.191453 | 7.5869575 | 0.023688743 | 0.276771487 | Up |  |
| ENSGALG00000012165 | 2.3328872 | 1.925804 | 0.024165522 | 0.276655672 | Up |  |
| ENSGALG00000005710 | 65.8496 | 54.36735 | 0.023454817 | 0.276434168 | Up |  |
| ENSGALG00000016578 | 30.208195 | 25.077919 | 0.04325508 | 0.268522346 | Up |  |
| ENSGALG00000026899 | 2.0745795 | 1.7284807 | 0.027382966 | 0.263314449 | Up |  |
| ENSGALG00000038561 | 1.199039 | 1 | 0.002504281 | 0.261878585 | Up |  |
| ENSGALG00000009841 | 4.3934026 | 3.6743422 | 0.046721067 | 0.257852716 | Up |  |
| ENSGALG00000008431 | 41.20121 | 34.494457 | 0.024365393 | 0.256322157 | Up |  |
| ENSGALG00000015271 | 6.733897 | 5.6409574 | 0.030135803 | 0.255501612 | Up |  |
| ENSGALG00000011492 | 9.929752 | 8.319957 | 0.029097749 | 0.255181614 | Up |  |
| ENSGALG00000011132 | 22.587513 | 18.926056 | 0.04360457 | 0.255151632 | Up |  |
| ENSGALG00000014152 | 21.522888 | 18.052795 | 0.022799984 | 0.253649458 | Up |  |
| ENSGALG00000017485 | 1.3877771 | 1.1653843 | 0.030542413 | 0.251970086 | Up |  |
| ENSGALG00000011847 | 15.12532 | 12.754509 | 0.03919776 | 0.245958303 | Up |  |
| ENSGALG00000050288 | 13.195209 | 11.1290245 | 0.029691525 | 0.245687061 | Up |  |
| ENSGALG00000017148 | 1.413918 | 1.1942493 | 0.036446568 | 0.243594422 | Up |  |
| ENSGALG00000009338 | 10.6073885 | 8.973222 | 0.01659259 | 0.241371504 | Up |  |
| ENSGALG00000011353 | 1.512421 | 1.2802624 | 0.015355501 | 0.240420254 | Up |  |
| ENSGALG00000004033 | 4.9330387 | 4.177695 | 0.001357473 | 0.239769436 | Up |  |
| ENSGALG00000006127 | 61.518738 | 52.249313 | 0.021131624 | 0.23561384 | Up |  |
| ENSGALG00000050980 | 5.0335464 | 4.2796593 | 0.022827204 | 0.234079264 | Up |  |
| ENSGALG00000010598 | 3.3280525 | 2.8299267 | 0.04203361 | 0.233913507 | Up |  |
| ENSGALG00000015708 | 12.395546 | 10.543615 | 0.041226484 | 0.233452224 | Up |  |
| ENSGALG00000008659 | 4.0690475 | 3.4622443 | 0.022934016 | 0.232983595 | Up |  |
| ENSGALG00000047617 | 25.711304 | 21.880096 | 0.016165031 | 0.232783713 | Up |  |
| ENSGALG00000006108 | 42.70224 | 36.37759 | 0.026280865 | 0.231261782 | Up |  |
| ENSGALG00000042150 | 1.2632265 | 1.0763646 | 0.02204023 | 0.230946493 | Up |  |
| ENSGALG00000025253 | 1.170977 | 1 | 0.03911749 | 0.227712739 | Up |  |
| ENSGALG00000012699 | 8.6819935 | 7.4203153 | 0.034748595 | 0.226545852 | Up |  |
| ENSGALG00000013911 | 17.174637 | 14.693157 | 0.039730612 | 0.225135197 | Up |  |
| ENSGALG00000009217 | 8.058502 | 6.8992352 | 0.014230308 | 0.224075236 | Up |  |
| ENSGALG00000038395 | 12.953419 | 11.094562 | 0.027836466 | 0.223480229 | Up |  |
| ENSGALG00000000551 | 13.274152 | 11.372749 | 0.024916852 | 0.223038678 | Up |  |
| ENSGALG00000002165 | 44.154358 | 37.82986 | 0.029951802 | 0.2230304 | Up |  |
| ENSGALG00000010610 | 21.751484 | 18.680885 | 0.041172057 | 0.219551029 | Up |  |
| ENSGALG00000012304 | 19.482779 | 16.740662 | 0.048891727 | 0.218842897 | Up |  |
| ENSGALG00000027198 | 20.187614 | 17.34806 | 0.019489873 | 0.218696069 | Up |  |
| ENSGALG00000013602 | 19.931404 | 17.163734 | 0.021863032 | 0.215679892 | Up |  |
| ENSGALG00000049811 | 52.159122 | 44.918934 | 0.036374763 | 0.215595894 | Up |  |
| ENSGALG00000010649 | 9.905417 | 8.536263 | 0.02509645 | 0.214613085 | Up |  |
| ENSGALG00000028691 | 9.133151 | 7.873867 | 0.008776745 | 0.214040342 | Up |  |
| ENSGALG00000030545 | 1.18172 | 1.0208377 | 0.02753566 | 0.211134725 | Up |  |
| ENSGALG00000047385 | 1.2692785 | 1.0976987 | 0.046961263 | 0.209526541 | Up |  |
| ENSGALG00000008562 | 1.212153 | 1.0495623 | 0.04802112 | 0.207784005 | Up |  |
| ENSGALG00000008096 | 20.510948 | 17.76628 | 0.010112496 | 0.20725254 | Up |  |
| ENSGALG00000010642 | 26.106197 | 22.64657 | 0.030402357 | 0.20509975 | Up |  |
| ENSGALG00000015086 | 12.603884 | 10.93396 | 0.013593086 | 0.205052379 | Up |  |
| ENSGALG00000015443 | 8.353005 | 7.255299 | 0.047250643 | 0.203260231 | Up | |
| ENSGALG00000005998 | 12.670501 | 11.006333 | 0.003590696 | 0.203139687 | Up |  |
| ENSGALG00000010284 | 18.531805 | 16.099817 | 0.015373908 | 0.202959117 | Up |  |
| ENSGALG00000005979 | 12.12182 | 10.549996 | 0.015665729 | 0.200363873 | Up |  |
| ENSGALG00000004750 | 8.3923645 | 7.3132806 | 0.043827694 | 0.198558622 | Up |  |
| ENSGALG00000032365 | 48.002632 | 41.853466 | 0.03870552 | 0.19776641 | Up |  |
| ENSGALG00000048304 | 1.183574 | 1.0321647 | 0.022830246 | 0.197476713 | Up |  |
| ENSGALG00000031663 | 138.76596 | 121.19813 | 0.037437703 | 0.195286272 | Up |  |
| ENSGALG00000010476 | 117.11054 | 102.398506 | 0.0035866 | 0.193676258 | Up |  |
| ENSGALG00000003392 | 29.46386 | 25.771324 | 0.041455474 | 0.19317979 | Up |  |
| ENSGALG00000031069 | 14.337589 | 12.556302 | 0.02681193 | 0.191390808 | Up |  |
| ENSGALG00000001146 | 17.660263 | 15.468613 | 0.022051567 | 0.191162985 | Up |  |
| ENSGALG00000050084 | 1.2897525 | 1.1302024 | 0.012455843 | 0.190513085 | Up |  |
| ENSGALG00000036892 | 1.199934 | 1.0516124 | 0.029825512 | 0.190351997 | Up |  |
| ENSGALG00000015322 | 2.6404254 | 2.3161297 | 0.007221904 | 0.189054337 | Up |  |
| ENSGALG00000019795 | 5.804558 | 5.093844 | 0.018511675 | 0.188431437 | Up |  |
| ENSGALG00000008128 | 42.5942 | 37.49343 | 0.034328565 | 0.184019181 | Up |  |
| ENSGALG00000039450 | 14.004248 | 12.352352 | 0.0464859 | 0.181078745 | Up |  |
| ENSGALG00000029047 | 16.24104 | 14.343697 | 0.044252966 | 0.179227101 | Up |  |
| ENSGALG00000052140 | 1.1318431 | 1 | 0.00552376 | 0.178673981 | Up |  |
| ENSGALG00000003812 | 21.94387 | 19.413683 | 0.042696677 | 0.176744141 | Up |  |
| ENSGALG00000002307 | 1.1291995 | 1 | 0.01736258 | 0.175300395 | Up |  |
| ENSGALG00000028685 | 3.620894 | 3.2099886 | 0.0330505 | 0.173777769 | Up |  |
| ENSGALG00000015184 | 10.255301 | 9.109342 | 0.04137942 | 0.170951085 | Up |  |
| ENSGALG00000053163 | 1.2401786 | 1.1049737 | 0.019795997 | 0.166535869 | Up |  |
| ENSGALG00000011932 | 13.100311 | 11.692676 | 0.024654265 | 0.163995917 | Up |  |
| ENSGALG00000048028 | 1.465867 | 1.3119277 | 0.013314241 | 0.160065996 | Up |  |
| ENSGALG00000005575 | 24.48291 | 21.913214 | 0.049929067 | 0.159973945 | Up |  |
| ENSGALG00000041993 | 34.238247 | 30.648705 | 0.007405473 | 0.159782721 | Up |  |
| ENSGALG00000029760 | 5.72946 | 5.1291213 | 0.00793348 | 0.159687481 | Up |  |
| ENSGALG00000001362 | 34.648296 | 31.031548 | 0.0236002 | 0.159048735 | Up |  |
| ENSGALG00000013685 | 10.7673025 | 9.655251 | 0.04466113 | 0.157271192 | Up |  |
| ENSGALG00000051525 | 1.1245251 | 1.0086864 | 0.0110556 | 0.156838152 | Up |  |
| ENSGALG00000014258 | 6.0040607 | 5.3858566 | 0.04511762 | 0.156762744 | Up |  |
| ENSGALG00000031294 | 5.7931347 | 5.2138543 | 0.031764228 | 0.151993943 | Up |  |
| ENSGALG00000009879 | 54.9698 | 49.521667 | 0.006120775 | 0.150579351 | Up |  |
| ENSGALG00000011024 | 8.489387 | 7.6511865 | 0.030830666 | 0.149976894 | Up |  |
| ENSGALG00000001963 | 6.0302 | 5.4464245 | 0.008025763 | 0.14689642 | Up |  |
| ENSGALG00000031117 | 6.8050985 | 6.15721 | 0.026319861 | 0.14433927 | Up |  |
| ENSGALG00000053474 | 1.1047075 | 1 | 0.004076529 | 0.143664429 | Up |  |
| ENSGALG00000050512 | 1.1427236 | 1.0352947 | 0.02910365 | 0.142434995 | Up |  |
| ENSGALG00000031821 | 39.784233 | 36.146877 | 0.030170018 | 0.138325778 | Up |  |
| ENSGALG00000039455 | 55.755096 | 50.78982 | 0.043114148 | 0.134564312 | Up |  |
| ENSGALG00000009018 | 63.041683 | 57.543762 | 0.010735963 | 0.131646508 | Up |  |
| ENSGALG00000037697 | 167.95956 | 153.7652 | 0.011711852 | 0.127384883 | Up |  |
| ENSGALG00000004297 | 1.112201 | 1.0199863 | 0.007966794 | 0.124867765 | Up |  |
| ENSGALG00000049735 | 1.1181619 | 1.0272154 | 0.029967375 | 0.122390356 | Up |  |
| ENSGALG00000054676 | 1.1265764 | 1.037934 | 0.029551135 | 0.118230446 | Up |  |
| ENSGALG00000050445 | 1.0984625 | 1.0139409 | 0.027394047 | 0.115512055 | Up |  |
| ENSGALG00000016894 | 63.909092 | 59.02368 | 0.03761234 | 0.114727318 | Up |  |
| ENSGALG00000050131 | 1.3334291 | 1.2325646 | 0.03685713 | 0.113477855 | Up |  |
| ENSGALG00000046781 | 1.078125 | 1 | 0.00898781 | 0.108524457 | Up |  |
| ENSGALG00000039130 | 19.896482 | 18.473825 | 0.02737699 | 0.107030756 | Up |  |
| ENSGALG00000017388 | 35.822617 | 33.306763 | 0.049975935 | 0.105055587 | Up |  |
| ENSGALG00000044243 | 1.0750855 | 1 | 0.005565934 | 0.1044514 | Up |  |
| ENSGALG00000032036 | 7.7811537 | 7.243187 | 0.04654757 | 0.103359455 | Up |  |
| ENSGALG00000040436 | 1.0806711 | 1.0075268 | 0.010897946 | 0.101109294 | Up |  |
| ENSGALG00000005279 | 3.0452895 | 2.8414106 | 0.022675144 | 0.09997206 | Up |  |
| ENSGALG00000001838 | 27.497625 | 25.735146 | 0.009183115 | 0.09556705 | Up |  |
| ENSGALG00000053448 | 1.0647135 | 1 | 0.026530977 | 0.090465273 | Up |  |
| ENSGALG00000016776 | 10.304501 | 9.691405 | 0.048767272 | 0.088496904 | Up |  |
| ENSGALG00000017235 | 1.073092 | 1.0114233 | 0.0381388 | 0.08538685 | Up |  |
| ENSGALG00000003228 | 18.3782 | 17.348612 | 0.03056702 | 0.08317523 | Up |  |
| ENSGALG00000048836 | 1.05784 | 1 | 0.02055323 | 0.081121434 | Up |  |
| ENSGALG00000040155 | 14.166051 | 13.391816 | 0.046414543 | 0.08108603 | Up |  |
| ENSGALG00000003398 | 30.253448 | 28.64154 | 0.033357475 | 0.078990511 | Up |  |
| ENSGALG00000053151 | 1.0557585 | 1 | 0.01433233 | 0.078279862 | Up |  |
| ENSGALG00000006639 | 1.052466 | 1 | 0.020135496 | 0.073773628 | Up |  |
| ENSGALG00000004618 | 39.31087 | 37.56462 | 0.04660191 | 0.065553785 | Up |  |
| ENSGALG00000054291 | 1.044528 | 1 | 0.023308398 | 0.062851166 | Up |  |
| ENSGALG00000049425 | 1.0400286 | 1 | 0.026536038 | 0.056623202 | Up |  |
| ENSGALG00000038155 | 33.458954 | 32.180546 | 0.026400976 | 0.05620354 | Up |  |
| ENSGALG00000050665 | 1.0456984 | 1.0072597 | 0.035772994 | 0.054031111 | Up |  |
| ENSGALG00000016470 | 1.0376295 | 1 | 0.026537754 | 0.053291401 | Up |  |
| ENSGALG00000031766 | 27.831623 | 26.963095 | 0.034518667 | 0.045738934 | Up |  |
| ENSGALG00000030985 | 1.0561144 | 1.0239539 | 0.022276666 | 0.044615354 | Up |  |
| ENSGALG00000051776 | 1.0103354 | 1.0292116 | 0.012409873 | -0.02670532 | Down |  |
| ENSGALG00000051577 | 1 | 1.0313207 | 0.004131641 | -0.044493024 | Down |  |
| ENSGALG00000053219 | 1 | 1.0340971 | 0.023920888 | -0.048371659 | Down |  |
| ENSGALG00000049414 | 1 | 1.0365993 | 0.015549283 | -0.051858325 | Down |  |
| ENSGALG00000027742 | 1 | 1.041464 | 0.041949563 | -0.058612971 | Down |  |
| ENSGALG00000050242 | 1.029583 | 1.0740243 | 0.0466477 | -0.060966497 | Down |  |
| ENSGALG00000011742 | 1 | 1.0487226 | 0.029148612 | -0.068633118 | Down |  |
| ENSGALG00000040528 | 11.852997 | 12.446372 | 0.04245226 | -0.070473385 | Down |  |
| ENSGALG00000049162 | 1 | 1.0502363 | 0.003323979 | -0.070713966 | Down |  |
| ENSGALG00000016199 | 1 | 1.0556756 | 0.03595191 | -0.078166575 | Down |  |
| ENSGALG00000015722 | 1 | 1.058764 | 0.019555751 | -0.082381046 | Down |  |
| ENSGALG00000050458 | 1 | 1.0631686 | 0.041608177 | -0.088370401 | Down |  |
| ENSGALG00000011222 | 1.1614699 | 1.2440109 | 0.028262459 | -0.09904736 | Down |  |
| ENSGALG00000003138 | 1 | 1.0725586 | 0.027334621 | -0.101056473 | Down |  |
| ENSGALG00000003385 | 25.211023 | 27.142647 | 0.03292359 | -0.106506762 | Down |  |
| ENSGALG00000053031 | 1 | 1.0773159 | 0.037334267 | -0.107441352 | Down |  |
| ENSGALG00000053630 | 1.0116146 | 1.0917281 | 0.036935817 | -0.109953827 | Down |  |
| ENSGALG00000002093 | 1 | 1.079763 | 0.02967001 | -0.110714686 | Down |  |
| ENSGALG00000037136 | 12.919252 | 13.955333 | 0.043184508 | -0.111294007 | Down |  |
| ENSGALG00000047257 | 1 | 1.0806837 | 0.006136008 | -0.11194433 | Down |  |
| ENSGALG00000040511 | 9.61541 | 10.477828 | 0.046425015 | -0.123919404 | Down |  |
| ENSGALG00000050703 | 1 | 1.090573 | 0.001690483 | -0.125086343 | Down |  |
| ENSGALG00000009253 | 47.852264 | 52.270523 | 0.020403827 | -0.127410411 | Down |  |
| ENSGALG00000000227 | 38.27717 | 41.993126 | 0.045880962 | -0.133669019 | Down |  |
| ENSGALG00000016642 | 1 | 1.0989383 | 0.026753942 | -0.136110388 | Down |  |
| ENSGALG00000050353 | 1 | 1.10079 | 0.038697187 | -0.138539269 | Down |  |
| ENSGALG00000000184 | 1 | 1.1017426 | 0.0370669 | -0.139787207 | Down |  |
| ENSGALG00000049984 | 1 | 1.10331 | 0.03956084 | -0.141838206 | Down |  |
| ENSGALG00000011619 | 19.089853 | 21.212954 | 0.012298555 | -0.152139543 | Down |  |
| ENSGALG00000000824 | 1.017175 | 1.1340283 | 0.049038097 | -0.156888734 | Down |  |
| ENSGALG00000034013 | 16.589447 | 18.53378 | 0.02714116 | -0.159891356 | Down |  |
| ENSGALG00000053821 | 1 | 1.1241307 | 0.030239234 | -0.168809784 | Down |  |
| ENSGALG00000043632 | 2.156097 | 2.4331944 | 0.021689314 | -0.174429504 | Down |  |
| ENSGALG00000054962 | 1.1802965 | 1.333191 | 0.028127348 | -0.175734162 | Down |  |
| ENSGALG00000008303 | 9.830753 | 11.11069 | 0.045660976 | -0.176574583 | Down |  |
| ENSGALG00000044904 | 1.4717015 | 1.6644274 | 0.04504741 | -0.177540859 | Down |  |
| ENSGALG00000051060 | 1.0198375 | 1.1534317 | 0.04364301 | -0.177593285 | Down |  |
| ENSGALG00000009604 | 4.9638915 | 5.6208534 | 0.038468175 | -0.179317606 | Down |  |
| ENSGALG00000036683 | 20.839504 | 23.701912 | 0.049011037 | -0.185682503 | Down |  |
| ENSGALG00000008898 | 1.059155 | 1.2051337 | 0.020815752 | -0.186279478 | Down |  |
| ENSGALG00000031734 | 2.4519105 | 2.795985 | 0.03184374 | -0.189450302 | Down |  |
| ENSGALG00000042647 | 48.753555 | 55.638382 | 6.23E-04 | -0.190573045 | Down |  |
| ENSGALG00000012179 | 8.622247 | 9.850373 | 0.007652743 | -0.192114464 | Down |  |
| ENSGALG00000040759 | 6.774534 | 7.7423024 | 0.04868407 | -0.192640947 | Down |  |
| ENSGALG00000032250 | 16.747662 | 19.16076 | 0.012740222 | -0.194195079 | Down |  |
| ENSGALG00000002450 | 15.213495 | 17.419968 | 0.04489273 | -0.195390352 | Down |  |
| ENSGALG00000006806 | 6.0218973 | 6.927843 | 0.006550009 | -0.202188132 | Down |  |
| ENSGALG00000051337 | 1.1337775 | 1.3107123 | 0.042602375 | -0.209213506 | Down |  |
| ENSGALG00000053222 | 1 | 1.159273 | 0.008072581 | -0.21322035 | Down |  |
| ENSGALG00000007714 | 1.124052 | 1.3072196 | 0.017669879 | -0.217792742 | Down |  |
| ENSGALG00000050091 | 1.145129 | 1.3322767 | 0.034016643 | -0.218383618 | Down |  |
| ENSGALG00000030925 | 7.8900547 | 9.196248 | 0.020450342 | -0.22101007 | Down |  |
| ENSGALG00000034973 | 14.202284 | 16.65048 | 0.046959613 | -0.229440806 | Down |  |
| ENSGALG00000023032 | 1.461568 | 1.714633 | 0.04023158 | -0.230382862 | Down |  |
| ENSGALG00000006608 | 9.093548 | 10.679065 | 0.04694057 | -0.231870137 | Down |  |
| ENSGALG00000012419 | 2.961504 | 3.479416 | 0.042201 | -0.232515144 | Down |  |
| ENSGALG00000003356 | 18.710165 | 22.007872 | 0.013940954 | -0.234197373 | Down |  |
| ENSGALG00000002591 | 45.793365 | 53.92087 | 0.03023318 | -0.235705193 | Down |  |
| ENSGALG00000054890 | 1.067482 | 1.260949 | 0.01345288 | -0.240298183 | Down |  |
| ENSGALG00000006220 | 5.9413776 | 7.033249 | 0.04386874 | -0.243393813 | Down |  |
| ENSGALG00000054931 | 20.289204 | 24.046804 | 0.03692798 | -0.245132896 | Down |  |
| ENSGALG00000038820 | 17.21584 | 20.512413 | 0.009914647 | -0.25276064 | Down |  |
| ENSGALG00000002009 | 4.152169 | 4.9493656 | 0.017375462 | -0.25337845 | Down |  |
| ENSGALG00000004504 | 1.7273226 | 2.062154 | 0.042836756 | -0.255614526 | Down |  |
| ENSGALG00000037380 | 1 | 1.195834 | 0.002568645 | -0.258017135 | Down |  |
| ENSGALG00000054952 | 3.767905 | 4.507412 | 0.020571016 | -0.258536735 | Down |  |
| ENSGALG00000016440 | 8.684071 | 10.395461 | 0.020683177 | -0.259510311 | Down |  |
| ENSGALG00000006638 | 9.08577 | 10.928352 | 0.016488986 | -0.266395168 | Down |  |
| ENSGALG00000054661 | 1.0136055 | 1.2235553 | 0.02880592 | -0.271583049 | Down |  |
| ENSGALG00000010360 | 9.304007 | 11.281159 | 0.048554454 | -0.277991207 | Down |  |
| ENSGALG00000034204 | 13.267193 | 16.133585 | 0.023410438 | -0.282203886 | Down |  |
| ENSGALG00000042275 | 4.1829376 | 5.1030545 | 0.029007008 | -0.286844573 | Down |  |
| ENSGALG00000049378 | 4.0745173 | 4.977185 | 0.048024748 | -0.288700856 | Down |  |
| ENSGALG00000042334 | 1 | 1.226736 | 0.03577906 | -0.294824807 | Down |  |
| ENSGALG00000054183 | 1.315191 | 1.6145247 | 0.042414054 | -0.295837181 | Down |  |
| ENSGALG00000047991 | 1.5987439 | 1.9654493 | 0.013869186 | -0.297920294 | Down |  |
| ENSGALG00000014252 | 1.4314575 | 1.7713423 | 0.018280365 | -0.307358193 | Down |  |
| ENSGALG00000054913 | 9.120995 | 11.356705 | 0.040804997 | -0.316281196 | Down |  |
| ENSGALG00000031274 | 25.322609 | 31.548225 | 0.028611701 | -0.317132783 | Down |  |
| ENSGALG00000035718 | 6.5298443 | 8.149756 | 0.003084265 | -0.319708274 | Down |  |
| ENSGALG00000034772 | 4.9305925 | 6.1864204 | 0.046939094 | -0.327343853 | Down |  |
| ENSGALG00000001330 | 63.5151 | 79.81333 | 0.045047358 | -0.329530101 | Down |  |
| ENSGALG00000026167 | 2.2466116 | 2.8247864 | 0.042655338 | -0.330391051 | Down |  |
| ENSGALG00000034301 | 1.250083 | 1.5805103 | 0.010402256 | -0.33836655 | Down |  |
| ENSGALG00000035117 | 7.9326477 | 10.039937 | 0.00497031 | -0.339875833 | Down |  |
| ENSGALG00000006968 | 1.3457456 | 1.7094517 | 0.037219044 | -0.345127952 | Down |  |
| ENSGALG00000050119 | 72.15256 | 91.75467 | 0.0363868 | -0.346731005 | Down |  |
| ENSGALG00000027418 | 18.883942 | 24.1306 | 0.034811668 | -0.353703831 | Down |  |
| ENSGALG00000048720 | 1.499998 | 1.9339463 | 0.00951687 | -0.366587159 | Down |  |
| ENSGALG00000026157 | 1.0871346 | 1.4041847 | 0.028693551 | -0.36920214 | Down |  |
| ENSGALG00000033093 | 1 | 1.2919827 | 0.040862624 | -0.369586752 | Down |  |
| ENSGALG00000053680 | 14.878412 | 19.22803 | 0.03430034 | -0.369990406 | Down |  |
| ENSGALG00000046023 | 28.633701 | 37.05927 | 0.047443766 | -0.372120308 | Down |  |
| ENSGALG00000025434 | 1 | 1.3025657 | 0.036119882 | -0.381356142 | Down |  |
| ENSGALG00000040178 | 1 | 1.3036953 | 0.02608273 | -0.382606722 | Down |  |
| ENSGALG00000050350 | 15.446821 | 20.203274 | 0.010191587 | -0.387279147 | Down |  |
| ENSGALG00000049622 | 8.4170885 | 11.013003 | 0.011406855 | -0.387814722 | Down |  |
| ENSGALG00000049502 | 1.07207 | 1.408102 | 0.014801805 | -0.393352735 | Down |  |
| ENSGALG00000049228 | 1 | 1.3198605 | 0.041825388 | -0.400385455 | Down |  |
| ENSGALG00000047207 | 1.1606026 | 1.5371131 | 0.022143854 | -0.405349256 | Down |  |
| ENSGALG00000032699 | 9.475891 | 12.618973 | 0.045447323 | -0.413260992 | Down |  |
| ENSGALG00000010468 | 3.27782 | 4.3895955 | 0.03451542 | -0.42135137 | Down |  |
| ENSGALG00000030792 | 9.2728405 | 12.44036 | 0.043771602 | -0.42394499 | Down |  |
| ENSGALG00000042553 | 5.900216 | 7.9789433 | 0.032258898 | -0.435429923 | Down |  |
| ENSGALG00000025211 | 1 | 1.352352 | 0.025482044 | -0.435470716 | Down |  |
| ENSGALG00000007014 | 1.9482136 | 2.645134 | 0.04075688 | -0.441188948 | Down |  |
| ENSGALG00000012128 | 5.066411 | 6.887858 | 0.037033867 | -0.443091284 | Down |  |
| ENSGALG00000041203 | 33.951504 | 46.38283 | 0.04430759 | -0.450115362 | Down |  |
| ENSGALG00000046585 | 11.509966 | 15.763547 | 0.029035917 | -0.453708624 | Down |  |
| ENSGALG00000023920 | 2.809366 | 3.872278 | 0.02709926 | -0.462937942 | Down |  |
| ENSGALG00000050717 | 1 | 1.3866571 | 0.03768545 | -0.471611074 | Down |  |
| ENSGALG00000029396 | 9.169165 | 12.764184 | 0.020301228 | -0.477239046 | Down |  |
| ENSGALG00000052798 | 1.7397965 | 2.4306896 | 0.030392393 | -0.482447105 | Down |  |
| ENSGALG00000047412 | 6.951292 | 9.747354 | 0.018923067 | -0.487729492 | Down |  |
| ENSGALG00000045218 | 1.9772505 | 2.822242 | 0.023440437 | -0.513346041 | Down |  |
| ENSGALG00000000639 | 7.8548765 | 11.284894 | 0.022049958 | -0.52273237 | Down |  |
| ENSGALG00000039628 | 4.188903 | 6.0465837 | 0.013452548 | -0.529547778 | Down |  |
| ENSGALG00000031238 | 43.69575 | 63.76044 | 0.011190773 | -0.54516862 | Down |  |
| ENSGALG00000015069 | 10.92594 | 16.036654 | 0.02113702 | -0.553615754 | Down |  |
| ENSGALG00000008985 | 48.112965 | 70.62637 | 0.025994722 | -0.553781238 | Down |  |
| ENSGALG00000054594 | 7.276002 | 11.136554 | 0.04894216 | -0.614085042 | Down |  |
| ENSGALG00000032889 | 4.329949 | 6.642403 | 0.019931316 | -0.617355223 | Down |  |
| ENSGALG00000025372 | 3.087586 | 4.770023 | 0.040531445 | -0.627516901 | Down |  |
| ENSGALG00000048260 | 1 | 1.5455817 | 0.0320279 | -0.628149918 | Down |  |
| ENSGALG00000054703 | 21.080463 | 33.143368 | 0.025655616 | -0.652813662 | Down |  |
| ENSGALG00000047528 | 1 | 1.580743 | 0.038698025 | -0.660602831 | Down |  |
| ENSGALG00000050174 | 3.7557416 | 5.9385204 | 0.037666246 | -0.661005717 | Down |  |
| ENSGALG00000047542 | 5.3767705 | 8.517907 | 0.008928129 | -0.663759085 | Down |  |
| ENSGALG00000051068 | 2.3911734 | 3.8141346 | 0.016823301 | -0.673637004 | Down |  |
| ENSGALG00000049767 | 1.509419 | 2.4241455 | 0.016013594 | -0.683482954 | Down |  |
| ENSGALG00000037082 | 5.0251093 | 8.386983 | 0.020216972 | -0.738996955 | Down |  |
| ENSGALG00000007762 | 20.375046 | 36.250736 | 0.03750806 | -0.83120697 | Down |  |
| ENSGALG00000017817 | 1 | 1.805551 | 0.014336773 | -0.852439172 | Down |  |
| ENSGALG00000044505 | 3.70147 | 6.734125 | 0.013303612 | -0.863392165 | Down |  |
| ENSGALG00000025620 | 1.7295365 | 3.2376604 | 0.042883843 | -0.904566208 | Down |  |
| ENSGALG00000026107 | 2.4070885 | 4.7971363 | 0.04112281 | -0.994884246 | Down |  |
| ENSGALG00000049827 | 1.0893564 | 2.2436397 | 0.03222866 | -1.042364985 | Down |  |

**Table S4.** List of DEGs in ovary samples between HEP and LEP chickens

| **GeneID** | **Mean-HEP** | **Mean-LEP** | **Adj p value** | **log2Ratio (LEP/HEP)** | **Type** |
| --- | --- | --- | --- | --- | --- |
| ENSGALG00000047299 | 7.0570908 | 1.8781067 | 0.011460568 | 1.909794544 | Up |
| ENSGALG00000025951 | 4.0315304 | 1.5506147 | 0.04863956 | 1.378487354 | Up |
| ENSGALG00000051980 | 23.22659 | 10.034197 | 0.02533583 | 1.210852193 | Up |
| ENSGALG00000009511 | 4.0636735 | 1.8204199 | 0.03245914 | 1.15851323 | Up |
| ENSGALG00000010718 | 21.698597 | 9.887422 | 0.025378965 | 1.13393545 | Up |
| ENSGALG00000035350 | 12.348608 | 6.171428 | 0.04057888 | 1.000672166 | Up |
| ENSGALG00000025454 | 3.0487945 | 1.5353254 | 0.009961984 | 0.989694455 | Up |
| ENSGALG00000042045 | 5.526184 | 2.812171 | 0.019059483 | 0.974599276 | Up |
| ENSGALG00000031116 | 2.221439 | 1.1797363 | 0.043849673 | 0.913030108 | Up |
| ENSGALG00000034189 | 4.6340785 | 2.4622042 | 0.0170526 | 0.912332068 | Up |
| ENSGALG00000003588 | 3.685539 | 1.9609853 | 0.013950832 | 0.910296904 | Up |
| ENSGALG00000043044 | 20.368437 | 11.431774 | 0.015244685 | 0.833285977 | Up |
| ENSGALG00000011990 | 15.724863 | 9.036019 | 0.028414216 | 0.799288239 | Up |
| ENSGALG00000027513 | 4.2160034 | 2.4302952 | 0.028544215 | 0.794744467 | Up |
| ENSGALG00000016628 | 3.7474184 | 2.1803353 | 0.029534744 | 0.781347049 | Up |
| ENSGALG00000001690 | 4.655388 | 2.7134447 | 0.029448628 | 0.778775906 | Up |
| ENSGALG00000019077 | 8.672808 | 5.073253 | 0.042081438 | 0.773588063 | Up |
| ENSGALG00000009836 | 23.339985 | 14.000222 | 0.039802257 | 0.73735393 | Up |
| ENSGALG00000028209 | 11.720589 | 7.0328426 | 0.043528873 | 0.736865238 | Up |
| ENSGALG00000015113 | 4.0959625 | 2.4631462 | 0.019417513 | 0.733700245 | Up |
| ENSGALG00000053052 | 2.0697265 | 1.245766 | 0.027235009 | 0.732407035 | Up |
| ENSGALG00000043130 | 2.0383396 | 1.2334083 | 0.008205661 | 0.724743974 | Up |
| ENSGALG00000047218 | 1.6636806 | 1.015977 | 7.54E-04 | 0.711510743 | Up |
| ENSGALG00000025330 | 2.015695 | 1.2361773 | 0.03619857 | 0.705391679 | Up |
| ENSGALG00000034586 | 8.637953 | 5.305409 | 0.048750423 | 0.703225492 | Up |
| ENSGALG00000009002 | 6.9063473 | 4.2901225 | 0.013803587 | 0.686904042 | Up |
| ENSGALG00000053051 | 1.979984 | 1.2390534 | 0.02577709 | 0.676250407 | Up |
| ENSGALG00000009981 | 5.2165613 | 3.26463 | 0.02500648 | 0.67617962 | Up |
| ENSGALG00000012978 | 5.263704 | 3.2955182 | 0.030821454 | 0.675573025 | Up |
| ENSGALG00000008193 | 11.24068 | 7.068138 | 0.03815543 | 0.669327201 | Up |
| ENSGALG00000023407 | 7.537704 | 4.772426 | 0.03641782 | 0.659402315 | Up |
| ENSGALG00000014878 | 13.472734 | 8.590993 | 0.04502684 | 0.649145843 | Up |
| ENSGALG00000013862 | 1.7535186 | 1.1430457 | 0.044807058 | 0.617369649 | Up |
| ENSGALG00000027758 | 1.9898245 | 1.2990217 | 0.048549056 | 0.615215661 | Up |
| ENSGALG00000006978 | 22.48328 | 14.883141 | 0.044150833 | 0.595173489 | Up |
| ENSGALG00000049045 | 1.6110971 | 1.076889 | 0.022554588 | 0.581173895 | Up |
| ENSGALG00000014346 | 18.228329 | 12.200465 | 0.021061515 | 0.57924618 | Up |
| ENSGALG00000009982 | 9.860683 | 6.6604257 | 0.02424926 | 0.566073188 | Up |
| ENSGALG00000015019 | 15.686351 | 10.596768 | 0.049326114 | 0.565885476 | Up |
| ENSGALG00000023142 | 10.871195 | 7.3476086 | 0.039829325 | 0.565163853 | Up |
| ENSGALG00000013095 | 15.770418 | 10.807916 | 0.03373792 | 0.545132533 | Up |
| ENSGALG00000016866 | 2.010207 | 1.3802651 | 0.021837866 | 0.542398686 | Up |
| ENSGALG00000049027 | 46.04437 | 31.69165 | 0.025348889 | 0.53892199 | Up |
| ENSGALG00000007266 | 10.546593 | 7.2640386 | 0.031140791 | 0.537933248 | Up |
| ENSGALG00000033391 | 2.524187 | 1.7454191 | 0.036494654 | 0.532245304 | Up |
| ENSGALG00000028180 | 1.590523 | 1.1029193 | 0.04366232 | 0.528174001 | Up |
| ENSGALG00000007800 | 5.530308 | 3.8351886 | 0.011176182 | 0.528062305 | Up |
| ENSGALG00000052717 | 1.6467215 | 1.1450653 | 0.040674694 | 0.524166708 | Up |
| ENSGALG00000010163 | 2.0620284 | 1.4349216 | 0.04432391 | 0.523092289 | Up |
| ENSGALG00000007996 | 18.07861 | 12.600833 | 0.020447852 | 0.52076465 | Up |
| ENSGALG00000030543 | 24.869267 | 17.338537 | 0.016407492 | 0.520381815 | Up |
| ENSGALG00000003716 | 2.9911962 | 2.0855067 | 0.03517706 | 0.520324596 | Up |
| ENSGALG00000014645 | 9.912627 | 6.9308 | 0.044929426 | 0.516245557 | Up |
| ENSGALG00000053091 | 2.4174445 | 1.6983153 | 0.029986536 | 0.509378442 | Up |
| ENSGALG00000032808 | 11.978973 | 8.41737 | 0.02878073 | 0.509062786 | Up |
| ENSGALG00000008306 | 31.250652 | 21.980444 | 0.031025626 | 0.507665761 | Up |
| ENSGALG00000037727 | 5.755438 | 4.0522685 | 0.013785345 | 0.506195955 | Up |
| ENSGALG00000032850 | 18.037594 | 12.708695 | 0.037799638 | 0.505191019 | Up |
| ENSGALG00000038971 | 5.453157 | 3.8625536 | 0.047911525 | 0.497536739 | Up |
| ENSGALG00000011106 | 15.826702 | 11.260253 | 0.031378925 | 0.491121412 | Up |
| ENSGALG00000017894 | 1.541031 | 1.0992603 | 0.047173142 | 0.487362833 | Up |
| ENSGALG00000000249 | 4.4084296 | 3.1450806 | 0.005209429 | 0.487167831 | Up |
| ENSGALG00000051378 | 10.850058 | 7.7681274 | 0.012852259 | 0.482063988 | Up |
| ENSGALG00000035207 | 3.111458 | 2.228219 | 0.048470948 | 0.481699738 | Up |
| ENSGALG00000010629 | 14.562309 | 10.4303465 | 0.044327423 | 0.481452042 | Up |
| ENSGALG00000014699 | 3.3576756 | 2.410676 | 0.025123993 | 0.478025089 | Up |
| ENSGALG00000052683 | 1.516206 | 1.0922577 | 0.013429785 | 0.473152503 | Up |
| ENSGALG00000004907 | 10.314354 | 7.455851 | 0.013361192 | 0.468208532 | Up |
| ENSGALG00000006929 | 16.81431 | 12.276067 | 0.030817587 | 0.453841152 | Up |
| ENSGALG00000007862 | 2.9196692 | 2.1370952 | 0.046464667 | 0.450153744 | Up |
| ENSGALG00000003849 | 2.5421205 | 1.864323 | 0.025514683 | 0.447380584 | Up |
| ENSGALG00000032651 | 1.412488 | 1.0367314 | 0.01383672 | 0.446196447 | Up |
| ENSGALG00000054073 | 2.0750325 | 1.5267147 | 0.040726934 | 0.442703444 | Up |
| ENSGALG00000002543 | 24.092854 | 17.760382 | 0.006777392 | 0.439942691 | Up |
| ENSGALG00000010384 | 3.3910089 | 2.508101 | 0.018238638 | 0.435119125 | Up |
| ENSGALG00000005673 | 11.951212 | 8.865276 | 0.032687705 | 0.430919481 | Up |
| ENSGALG00000041114 | 17.870485 | 13.267377 | 0.04419372 | 0.429695615 | Up |
| ENSGALG00000037170 | 1.7050805 | 1.2698447 | 0.02553571 | 0.425187785 | Up |
| ENSGALG00000010763 | 15.372646 | 11.507427 | 0.031579673 | 0.417800218 | Up |
| ENSGALG00000022887 | 4.9384737 | 3.6984673 | 0.043607596 | 0.417137707 | Up |
| ENSGALG00000041680 | 1.7237685 | 1.2930477 | 0.016293647 | 0.414790539 | Up |
| ENSGALG00000033340 | 17.158003 | 12.876069 | 0.047329072 | 0.414189436 | Up |
| ENSGALG00000040363 | 9.466707 | 7.129803 | 0.04085856 | 0.409000456 | Up |
| ENSGALG00000002347 | 9.252338 | 6.9695687 | 0.041513216 | 0.40874859 | Up |
| ENSGALG00000008889 | 26.006653 | 19.59654 | 0.03530877 | 0.408281787 | Up |
| ENSGALG00000014624 | 2.8358312 | 2.1434886 | 0.04304454 | 0.403810916 | Up |
| ENSGALG00000017832 | 1.453003 | 1.0983626 | 0.010697576 | 0.403683275 | Up |
| ENSGALG00000043133 | 13.009571 | 9.880886 | 0.032940995 | 0.396861073 | Up |
| ENSGALG00000052822 | 1.5051885 | 1.1432987 | 0.006734287 | 0.396741798 | Up |
| ENSGALG00000034964 | 4.4720592 | 3.4119952 | 0.049575623 | 0.390323669 | Up |
| ENSGALG00000046757 | 1.6795955 | 1.2829362 | 0.044876214 | 0.388664401 | Up |
| ENSGALG00000008686 | 16.857498 | 12.881938 | 0.045899805 | 0.388040773 | Up |
| ENSGALG00000009932 | 9.77882 | 7.4742455 | 0.040992748 | 0.387732436 | Up |
| ENSGALG00000036201 | 15.211771 | 11.641032 | 0.03139606 | 0.385969164 | Up |
| ENSGALG00000029996 | 28.293724 | 21.67608 | 0.046795614 | 0.384378199 | Up |
| ENSGALG00000041098 | 3.3933296 | 2.6039016 | 0.039510958 | 0.382026637 | Up |
| ENSGALG00000015576 | 8.795633 | 6.753498 | 0.025394196 | 0.381152463 | Up |
| ENSGALG00000023089 | 12.038105 | 9.296622 | 0.04748443 | 0.372829803 | Up |
| ENSGALG00000016852 | 22.971556 | 17.773094 | 0.027032698 | 0.37015373 | Up |
| ENSGALG00000016855 | 70.2148 | 54.36964 | 0.043912675 | 0.368973881 | Up |
| ENSGALG00000003663 | 9.36384 | 7.259084 | 0.04170979 | 0.367312773 | Up |
| ENSGALG00000043765 | 15.696549 | 12.1839285 | 0.046425097 | 0.365468026 | Up |
| ENSGALG00000049271 | 1.585701 | 1.2309653 | 0.03921153 | 0.365330668 | Up |
| ENSGALG00000047100 | 1.7174435 | 1.3344587 | 0.049941033 | 0.364007982 | Up |
| ENSGALG00000051831 | 2.259416 | 1.7576493 | 0.034198407 | 0.362302681 | Up |
| ENSGALG00000013616 | 1.4064264 | 1.0977187 | 0.006774169 | 0.357525658 | Up |
| ENSGALG00000002942 | 18.350819 | 14.329971 | 0.025854807 | 0.356808762 | Up |
| ENSGALG00000000990 | 229.67255 | 179.36584 | 0.037855513 | 0.356673282 | Up |
| ENSGALG00000018302 | 1.350263 | 1.0548726 | 0.011482608 | 0.356171667 | Up |
| ENSGALG00000038377 | 5.3408318 | 4.1767926 | 0.03231536 | 0.354668942 | Up |
| ENSGALG00000052679 | 1.9313226 | 1.5119737 | 0.041515235 | 0.353156121 | Up |
| ENSGALG00000012763 | 30.594025 | 23.962698 | 0.024236808 | 0.35245957 | Up |
| ENSGALG00000025450 | 1.341156 | 1.0521387 | 0.031354755 | 0.350152155 | Up |
| ENSGALG00000037253 | 1.614326 | 1.2673358 | 0.04513576 | 0.349133109 | Up |
| ENSGALG00000003487 | 80.15502 | 63.159637 | 0.037528437 | 0.343789998 | Up |
| ENSGALG00000032780 | 10.765451 | 8.489432 | 0.04511015 | 0.342668824 | Up |
| ENSGALG00000017023 | 2.0755367 | 1.6378874 | 0.023272248 | 0.341648263 | Up |
| ENSGALG00000006108 | 48.63575 | 38.412815 | 0.014413916 | 0.340429473 | Up |
| ENSGALG00000014516 | 7.1633935 | 5.6655602 | 0.012289889 | 0.33842458 | Up |
| ENSGALG00000030024 | 20.291166 | 16.050764 | 0.009334873 | 0.3382098 | Up |
| ENSGALG00000014916 | 26.231483 | 20.798485 | 0.03592281 | 0.334820929 | Up |
| ENSGALG00000039585 | 1.310915 | 1.0428177 | 0.03344361 | 0.330087169 | Up |
| ENSGALG00000009856 | 162.96457 | 129.85075 | 0.018415973 | 0.327703996 | Up |
| ENSGALG00000012072 | 15.03926 | 11.987515 | 0.026035534 | 0.327200961 | Up |
| ENSGALG00000016618 | 4.8774023 | 3.895258 | 0.04958215 | 0.324394087 | Up |
| ENSGALG00000054661 | 2.896957 | 2.3187492 | 0.03251673 | 0.321191487 | Up |
| ENSGALG00000009627 | 5.783252 | 4.632975 | 0.034947235 | 0.31994207 | Up |
| ENSGALG00000023920 | 4.573896 | 3.6671429 | 0.032096896 | 0.318767077 | Up |
| ENSGALG00000054527 | 3.0420914 | 2.4408848 | 0.04909045 | 0.317659292 | Up |
| ENSGALG00000042651 | 25.050182 | 20.114504 | 0.03652709 | 0.316584922 | Up |
| ENSGALG00000053439 | 1.282272 | 1.0305357 | 0.02463367 | 0.31530784 | Up |
| ENSGALG00000027907 | 63.26416 | 50.923603 | 0.032504205 | 0.313053927 | Up |
| ENSGALG00000003545 | 1.5999765 | 1.2938176 | 0.009689776 | 0.306416472 | Up |
| ENSGALG00000010869 | 11.584751 | 9.371047 | 0.022024715 | 0.305944885 | Up |
| ENSGALG00000020057 | 9.466725 | 7.6622124 | 0.041292515 | 0.305104395 | Up |
| ENSGALG00000002536 | 19.893126 | 16.117329 | 0.042574618 | 0.303657322 | Up |
| ENSGALG00000030460 | 7.943227 | 6.4488525 | 0.01389074 | 0.300682761 | Up |
| ENSGALG00000053199 | 1.451519 | 1.1851699 | 0.03903205 | 0.292469565 | Up |
| ENSGALG00000007151 | 15.255821 | 12.4726515 | 0.012031513 | 0.290591628 | Up |
| ENSGALG00000005351 | 2.0181 | 1.6566867 | 0.037741885 | 0.284696867 | Up |
| ENSGALG00000002499 | 12.222975 | 10.037099 | 0.01699741 | 0.284253121 | Up |
| ENSGALG00000011248 | 6.8323736 | 5.6222405 | 0.012360992 | 0.281241696 | Up |
| ENSGALG00000016024 | 4.2801514 | 3.527588 | 0.04357498 | 0.278979756 | Up |
| ENSGALG00000007125 | 13.726391 | 11.325531 | 0.013470716 | 0.277373663 | Up |
| ENSGALG00000037596 | 1.480244 | 1.2263752 | 0.028586054 | 0.271434578 | Up |
| ENSGALG00000047666 | 1.4715745 | 1.2212397 | 0.04790078 | 0.269014188 | Up |
| ENSGALG00000047431 | 1.2533801 | 1.0403917 | 0.034351017 | 0.268697198 | Up |
| ENSGALG00000053089 | 1.2141016 | 1.0086083 | 0.003862623 | 0.267523153 | Up |
| ENSGALG00000033186 | 16.862984 | 14.022319 | 0.04366232 | 0.266134891 | Up |
| ENSGALG00000005002 | 144.35892 | 120.13708 | 0.03186294 | 0.264978751 | Up |
| ENSGALG00000012418 | 2.592793 | 2.161864 | 0.032917388 | 0.262231263 | Up |
| ENSGALG00000011704 | 21.190056 | 17.676321 | 0.03381836 | 0.261569365 | Up |
| ENSGALG00000035673 | 1.3696275 | 1.1440264 | 0.031461954 | 0.25966323 | Up |
| ENSGALG00000031348 | 72.55845 | 60.73972 | 0.009202773 | 0.256503379 | Up |
| ENSGALG00000000235 | 63.298973 | 53.00716 | 0.03757748 | 0.255994846 | Up |
| ENSGALG00000020626 | 11.761808 | 9.870374 | 0.038971394 | 0.252933189 | Up |
| ENSGALG00000052354 | 44.18888 | 37.12047 | 0.031065177 | 0.251468388 | Up |
| ENSGALG00000037094 | 1.468387 | 1.233891 | 0.03139075 | 0.251017292 | Up |
| ENSGALG00000052771 | 1.6774089 | 1.412446 | 0.042232953 | 0.248038704 | Up |
| ENSGALG00000019178 | 6.688596 | 5.642847 | 0.040250026 | 0.245280174 | Up |
| ENSGALG00000001364 | 132.69626 | 112.00468 | 7.61E-04 | 0.244568694 | Up |
| ENSGALG00000015385 | 4.6086063 | 3.89054 | 0.033947207 | 0.244360115 | Up |
| ENSGALG00000003392 | 41.116547 | 34.897 | 0.035936873 | 0.236616094 | Up |
| ENSGALG00000036301 | 9.881516 | 8.400092 | 0.041945577 | 0.234327265 | Up |
| ENSGALG00000016739 | 26.521774 | 22.546928 | 0.005085752 | 0.234246397 | Up |
| ENSGALG00000007700 | 64.84109 | 55.145096 | 0.007177451 | 0.233675749 | Up |
| ENSGALG00000007757 | 28.92696 | 24.648829 | 0.017869936 | 0.230895605 | Up |
| ENSGALG00000015572 | 5.5846686 | 4.7841983 | 0.012455295 | 0.223194483 | Up |
| ENSGALG00000009684 | 1.3744155 | 1.1785313 | 0.039555177 | 0.221828137 | Up |
| ENSGALG00000011908 | 65.15286 | 56.013103 | 0.04070066 | 0.218064157 | Up |
| ENSGALG00000037968 | 50.402725 | 43.50098 | 0.045777608 | 0.212453832 | Up |
| ENSGALG00000000106 | 16.416243 | 14.185803 | 0.01490219 | 0.210676174 | Up |
| ENSGALG00000014965 | 1.292205 | 1.1167331 | 0.019482836 | 0.210550541 | Up |
| ENSGALG00000042764 | 12.595167 | 10.888196 | 0.041578837 | 0.210105309 | Up |
| ENSGALG00000013810 | 19.57613 | 16.942507 | 0.043225106 | 0.20844822 | Up |
| ENSGALG00000009551 | 41.754322 | 36.206333 | 0.049599353 | 0.205683472 | Up |
| ENSGALG00000050181 | 1.252378 | 1.0895687 | 0.045606554 | 0.200912906 | Up |
| ENSGALG00000039705 | 31.412912 | 27.339224 | 0.019103032 | 0.200385394 | Up |
| ENSGALG00000002487 | 31.844276 | 27.756165 | 0.038282294 | 0.198225824 | Up |
| ENSGALG00000051915 | 1.1816435 | 1.0309223 | 0.04365136 | 0.196859241 | Up |
| ENSGALG00000041795 | 13.389261 | 11.693614 | 0.00872939 | 0.195355461 | Up |
| ENSGALG00000053919 | 1.2049545 | 1.057768 | 0.017407706 | 0.187955434 | Up |
| ENSGALG00000005256 | 5.470645 | 4.809725 | 0.035512283 | 0.185756531 | Up |
| ENSGALG00000025253 | 1.159188 | 1.0201803 | 0.021776512 | 0.184290418 | Up |
| ENSGALG00000048961 | 1.606418 | 1.423986 | 0.017451791 | 0.173912378 | Up |
| ENSGALG00000008092 | 14.773191 | 13.100815 | 0.026879793 | 0.173324917 | Up |
| ENSGALG00000036556 | 21.20393 | 18.867186 | 0.040426325 | 0.168452419 | Up |
| ENSGALG00000055102 | 1.114737 | 1 | 0.032785065 | 0.156703375 | Up |
| ENSGALG00000043976 | 1.1379654 | 1.0241 | 0.042241972 | 0.152100096 | Up |
| ENSGALG00000014684 | 10.643946 | 9.643728 | 0.03834185 | 0.142370231 | Up |
| ENSGALG00000054658 | 1.198242 | 1.0906286 | 0.03490766 | 0.135759414 | Up |
| ENSGALG00000046141 | 1.1060835 | 1.0093373 | 0.018931307 | 0.132051927 | Up |
| ENSGALG00000001419 | 28.024792 | 25.745989 | 0.02305616 | 0.122355975 | Up |
| ENSGALG00000008241 | 12.058248 | 11.082694 | 0.031253096 | 0.12171169 | Up |
| ENSGALG00000036938 | 1.8154299 | 1.6697422 | 0.042743042 | 0.120685849 | Up |
| ENSGALG00000051906 | 1.1015725 | 1.013255 | 0.031685356 | 0.120567155 | Up |
| ENSGALG00000007917 | 1.2873416 | 1.1863624 | 0.025098871 | 0.117850148 | Up |
| ENSGALG00000003302 | 1.0821555 | 1 | 0.03162482 | 0.113907822 | Up |
| ENSGALG00000009639 | 1.1461954 | 1.0626516 | 0.018875435 | 0.109184338 | Up |
| ENSGALG00000049255 | 1.15326 | 1.0736717 | 0.012763911 | 0.103164879 | Up |
| ENSGALG00000007307 | 29.291359 | 27.416626 | 0.033622146 | 0.095424092 | Up |
| ENSGALG00000046517 | 1.1121805 | 1.0453486 | 0.043601558 | 0.089406819 | Up |
| ENSGALG00000048518 | 1.0843501 | 1.0194377 | 0.049817786 | 0.089057017 | Up |
| ENSGALG00000001030 | 39.603783 | 37.245502 | 0.021333486 | 0.088572038 | Up |
| ENSGALG00000016579 | 32.182182 | 30.512732 | 0.02174727 | 0.076850787 | Up |
| ENSGALG00000049988 | 1.057703 | 1.006116 | 0.013766168 | 0.07213793 | Up |
| ENSGALG00000009297 | 8.856611 | 8.451546 | 0.032593668 | 0.067539484 | Up |
| ENSGALG00000048525 | 1.0487665 | 1.0017463 | 0.004048511 | 0.066176327 | Up |
| ENSGALG00000009909 | 1.040673 | 1 | 0.007512619 | 0.057516817 | Up |
| ENSGALG00000051811 | 1.0340405 | 1 | 1.59E-04 | 0.048292692 | Up |
| ENSGALG00000049868 | 1.0273819 | 1 | 0.007509235 | 0.038972562 | Up |
| ENSGALG00000054614 | 1.0232675 | 1 | 0.007511283 | 0.03318334 | Up |
| ENSGALG00000051916 | 1.008493 | 1 | 0.007492296 | 0.01220107 | Up |
| ENSGALG00000049991 | 1.008493 | 1 | 0.007492296 | 0.01220107 | Up |
| ENSGALG00000013369 | 49.869534 | 51.379684 | 0.030299135 | -0.043039295 | Down |
| ENSGALG00000053271 | 1.0900095 | 1.1242981 | 0.021839535 | -0.044683898 | Down |
| ENSGALG00000012754 | 1 | 1.0326656 | 0.02358709 | -0.046373153 | Down |
| ENSGALG00000053077 | 1 | 1.038165 | 0.020798335 | -0.054035756 | Down |
| ENSGALG00000015494 | 1 | 1.0393947 | 0.04732926 | -0.055743608 | Down |
| ENSGALG00000006284 | 65.6449 | 69.2293 | 0.010559916 | -0.076699829 | Down |
| ENSGALG00000048326 | 1 | 1.0625488 | 0.0336116 | -0.087529102 | Down |
| ENSGALG00000048753 | 1 | 1.063836 | 0.04374115 | -0.089275763 | Down |
| ENSGALG00000049344 | 1 | 1.065627 | 0.04098338 | -0.091702542 | Down |
| ENSGALG00000027742 | 1 | 1.0702323 | 0.03646167 | -0.097923976 | Down |
| ENSGALG00000046870 | 1 | 1.0735167 | 0.03536509 | -0.102344634 | Down |
| ENSGALG00000040023 | 22.263592 | 23.908625 | 0.0275543 | -0.102844787 | Down |
| ENSGALG00000047485 | 1 | 1.07793 | 0.00184881 | -0.108263494 | Down |
| ENSGALG00000053715 | 1 | 1.0785513 | 0.021210901 | -0.109094798 | Down |
| ENSGALG00000037393 | 1 | 1.081254 | 0.005185545 | -0.11270547 | Down |
| ENSGALG00000018415 | 1 | 1.0822347 | 0.003988642 | -0.114013405 | Down |
| ENSGALG00000000722 | 1 | 1.0837764 | 0.035207342 | -0.116067137 | Down |
| ENSGALG00000050892 | 1 | 1.0840473 | 0.013752899 | -0.116427707 | Down |
| ENSGALG00000003194 | 37.989246 | 41.211838 | 0.028620366 | -0.117467729 | Down |
| ENSGALG00000007199 | 19.367817 | 21.019714 | 0.04373651 | -0.118081687 | Down |
| ENSGALG00000002016 | 14.001276 | 15.243764 | 0.009833381 | -0.122660865 | Down |
| ENSGALG00000047609 | 1 | 1.0914787 | 0.040043324 | -0.126283977 | Down |
| ENSGALG00000054205 | 7.4497824 | 8.134181 | 0.037195653 | -0.126798807 | Down |
| ENSGALG00000002463 | 15.191923 | 16.673166 | 0.045540407 | -0.13422358 | Down |
| ENSGALG00000054285 | 1.0351605 | 1.1442933 | 0.026878726 | -0.144602412 | Down |
| ENSGALG00000012703 | 20.92669 | 23.187075 | 0.04025648 | -0.147976702 | Down |
| ENSGALG00000007707 | 35.83487 | 39.761307 | 0.030909609 | -0.15000106 | Down |
| ENSGALG00000015822 | 1.1622655 | 1.29144 | 0.038646657 | -0.152040952 | Down |
| ENSGALG00000019738 | 1 | 1.1139944 | 0.04000885 | -0.15574198 | Down |
| ENSGALG00000005062 | 1.0165789 | 1.1351477 | 0.03314633 | -0.159157835 | Down |
| ENSGALG00000054819 | 1 | 1.1169494 | 0.006422409 | -0.15956383 | Down |
| ENSGALG00000050102 | 1 | 1.1173244 | 0.03695797 | -0.160048114 | Down |
| ENSGALG00000042415 | 17.71191 | 19.793846 | 0.0459429 | -0.160332163 | Down |
| ENSGALG00000026704 | 15.009074 | 16.895584 | 0.039863084 | -0.170811247 | Down |
| ENSGALG00000053319 | 1 | 1.1262671 | 0.04142494 | -0.17154901 | Down |
| ENSGALG00000010463 | 78.843994 | 88.84021 | 0.032005757 | -0.172211942 | Down |
| ENSGALG00000053421 | 1 | 1.1274563 | 0.002801514 | -0.173071516 | Down |
| ENSGALG00000047334 | 1.0908675 | 1.2309906 | 0.042362764 | -0.174343867 | Down |
| ENSGALG00000030728 | 1 | 1.1298 | 0.011687218 | -0.176067406 | Down |
| ENSGALG00000038084 | 1 | 1.1310067 | 0.009851289 | -0.177607476 | Down |
| ENSGALG00000051502 | 1 | 1.131076 | 0.022567617 | -0.177695871 | Down |
| ENSGALG00000050617 | 1 | 1.1334673 | 0.039638467 | -0.180742771 | Down |
| ENSGALG00000051176 | 1.082646 | 1.235693 | 0.04394835 | -0.190758767 | Down |
| ENSGALG00000004363 | 25.691273 | 29.44963 | 0.020421132 | -0.196971131 | Down |
| ENSGALG00000050600 | 1 | 1.1488557 | 0.039071128 | -0.200197602 | Down |
| ENSGALG00000035252 | 1099.0674 | 1267.6376 | 0.04575662 | -0.205862496 | Down |
| ENSGALG00000048351 | 1 | 1.1543177 | 0.041609727 | -0.207040348 | Down |
| ENSGALG00000027791 | 1 | 1.1590294 | 0.041043866 | -0.212917162 | Down |
| ENSGALG00000054382 | 1 | 1.1592194 | 0.044111185 | -0.213153644 | Down |
| ENSGALG00000001955 | 1.0148959 | 1.1770653 | 0.047964763 | -0.213862604 | Down |
| ENSGALG00000004332 | 1.262622 | 1.4645947 | 0.035164386 | -0.214078686 | Down |
| ENSGALG00000032002 | 1112.0801 | 1293.3481 | 0.032355018 | -0.217849919 | Down |
| ENSGALG00000052147 | 1 | 1.1657057 | 0.027568517 | -0.221203604 | Down |
| ENSGALG00000051350 | 1.1335995 | 1.323966 | 0.04722938 | -0.223955047 | Down |
| ENSGALG00000005019 | 1 | 1.1684209 | 0.00166987 | -0.22456007 | Down |
| ENSGALG00000047049 | 1 | 1.1705056 | 0.031443566 | -0.227131837 | Down |
| ENSGALG00000003400 | 38.84472 | 45.46831 | 0.046979193 | -0.227142869 | Down |
| ENSGALG00000016586 | 50.60437 | 59.322063 | 0.031684685 | -0.229306794 | Down |
| ENSGALG00000054544 | 1.502684 | 1.7639898 | 0.014406121 | -0.231300563 | Down |
| ENSGALG00000047472 | 17.140259 | 20.126884 | 0.041571353 | -0.231734925 | Down |
| ENSGALG00000049686 | 1 | 1.1759677 | 0.015191603 | -0.233848435 | Down |
| ENSGALG00000051662 | 1 | 1.1803716 | 0.017806001 | -0.239241115 | Down |
| ENSGALG00000041037 | 81.36093 | 96.37791 | 0.012730526 | -0.244366346 | Down |
| ENSGALG00000052955 | 1 | 1.186525 | 0.033826582 | -0.246742498 | Down |
| ENSGALG00000000507 | 1.24617 | 1.479932 | 0.018974174 | -0.248029997 | Down |
| ENSGALG00000000329 | 19.336653 | 22.980272 | 0.04618874 | -0.249057775 | Down |
| ENSGALG00000037256 | 4.095736 | 4.8770666 | 0.04122571 | -0.251890947 | Down |
| ENSGALG00000047847 | 9.298591 | 11.086464 | 0.041870534 | -0.253715266 | Down |
| ENSGALG00000049365 | 1.2985445 | 1.55015 | 0.025274709 | -0.25551237 | Down |
| ENSGALG00000015327 | 5.311705 | 6.3464336 | 0.03344861 | -0.256771067 | Down |
| ENSGALG00000028347 | 1.4954995 | 1.7869587 | 0.037483387 | -0.256878863 | Down |
| ENSGALG00000015854 | 24.877287 | 29.761572 | 0.037194822 | -0.258621571 | Down |
| ENSGALG00000036550 | 11.693548 | 13.9899 | 0.022941224 | -0.258672918 | Down |
| ENSGALG00000014442 | 1952.5681 | 2339.1802 | 0.030681532 | -0.260630138 | Down |
| ENSGALG00000005948 | 974.8894 | 1173.2947 | 0.030356454 | -0.267254964 | Down |
| ENSGALG00000002959 | 15.934237 | 19.192606 | 0.003816818 | -0.268420678 | Down |
| ENSGALG00000006408 | 11.878363 | 14.316892 | 0.005373108 | -0.269382311 | Down |
| ENSGALG00000012552 | 15.750956 | 18.990633 | 0.03489561 | -0.269848599 | Down |
| ENSGALG00000046747 | 1.1081314 | 1.337266 | 0.017640203 | -0.271157502 | Down |
| ENSGALG00000017824 | 1.353626 | 1.6365887 | 0.03646005 | -0.273862611 | Down |
| ENSGALG00000029897 | 256.90955 | 311.0872 | 0.01787823 | -0.276058515 | Down |
| ENSGALG00000054567 | 1.032906 | 1.264413 | 0.047927015 | -0.291758806 | Down |
| ENSGALG00000007241 | 29.481718 | 36.1517 | 0.032683264 | -0.294242893 | Down |
| ENSGALG00000053758 | 1.1835635 | 1.4558934 | 0.030480219 | -0.298767615 | Down |
| ENSGALG00000011615 | 1.575105 | 1.9448851 | 0.044523995 | -0.304236921 | Down |
| ENSGALG00000047301 | 1.231397 | 1.5210937 | 0.046183065 | -0.304813068 | Down |
| ENSGALG00000048196 | 1 | 1.240129 | 0.016928574 | -0.3104902 | Down |
| ENSGALG00000014938 | 1.6661706 | 2.0663896 | 0.023095433 | -0.310576161 | Down |
| ENSGALG00000006022 | 70.087875 | 87.18222 | 0.029184034 | -0.314869057 | Down |
| ENSGALG00000002634 | 32.27497 | 40.178295 | 0.04950128 | -0.316000589 | Down |
| ENSGALG00000037067 | 261.0431 | 327.33206 | 0.04989406 | -0.326466886 | Down |
| ENSGALG00000025785 | 1.1106935 | 1.3967043 | 0.006974046 | -0.330565862 | Down |
| ENSGALG00000005749 | 170.97086 | 216.18819 | 0.029447667 | -0.338537258 | Down |
| ENSGALG00000002551 | 2.349671 | 2.978587 | 0.027936181 | -0.342169333 | Down |
| ENSGALG00000035604 | 22.752338 | 29.003809 | 0.047825716 | -0.350227576 | Down |
| ENSGALG00000001054 | 40.90281 | 52.249924 | 0.04449114 | -0.35322898 | Down |
| ENSGALG00000004416 | 17.417679 | 22.26652 | 0.046406444 | -0.354323709 | Down |
| ENSGALG00000008806 | 497.03613 | 640.34015 | 0.04470541 | -0.365487745 | Down |
| ENSGALG00000039159 | 1 | 1.2922263 | 0.041558098 | -0.369858743 | Down |
| ENSGALG00000051861 | 1.9709435 | 2.5530708 | 0.040405545 | -0.373347126 | Down |
| ENSGALG00000049957 | 1.1114635 | 1.442756 | 0.027285952 | -0.376366759 | Down |
| ENSGALG00000035976 | 1.9329945 | 2.533028 | 0.010048246 | -0.390025492 | Down |
| ENSGALG00000001718 | 56.76397 | 74.48431 | 0.025693078 | -0.391961063 | Down |
| ENSGALG00000053521 | 1 | 1.3275727 | 0.014556255 | -0.408790867 | Down |
| ENSGALG00000042375 | 2.1555533 | 2.8691928 | 0.03841883 | -0.412586679 | Down |
| ENSGALG00000025899 | 1 | 1.3375969 | 0.013216394 | -0.419643409 | Down |
| ENSGALG00000029820 | 9.807304 | 13.263473 | 0.0334432 | -0.435530087 | Down |
| ENSGALG00000051568 | 1.3529615 | 1.8430084 | 0.035598174 | -0.44594186 | Down |
| ENSGALG00000050197 | 1.316184 | 1.7966094 | 0.03227721 | -0.448915598 | Down |
| ENSGALG00000003337 | 135.96114 | 185.67146 | 0.03394455 | -0.449557709 | Down |
| ENSGALG00000027712 | 1.280102 | 1.7529005 | 0.041008763 | -0.453485336 | Down |
| ENSGALG00000042222 | 7.415995 | 10.156526 | 0.04590598 | -0.453694842 | Down |
| ENSGALG00000024701 | 1 | 1.3718656 | 0.031691674 | -0.456139149 | Down |
| ENSGALG00000025382 | 15.753601 | 21.672903 | 0.029788023 | -0.460210768 | Down |
| ENSGALG00000052787 | 1 | 1.4013247 | 0.0201593 | -0.48679128 | Down |
| ENSGALG00000036780 | 2.4040456 | 3.3881 | 0.03378902 | -0.495012195 | Down |
| ENSGALG00000010193 | 10.86133 | 15.325368 | 0.04482616 | -0.496720941 | Down |
| ENSGALG00000025538 | 1 | 1.415401 | 0.007145951 | -0.501210844 | Down |
| ENSGALG00000002683 | 39.219433 | 55.59175 | 0.038475197 | -0.50330212 | Down |
| ENSGALG00000050023 | 2.178338 | 3.108721 | 0.024583882 | -0.513093318 | Down |
| ENSGALG00000037322 | 17.847357 | 25.62575 | 0.02432375 | -0.521883787 | Down |
| ENSGALG00000050115 | 1.2913179 | 1.883759 | 0.026250176 | -0.544770194 | Down |
| ENSGALG00000030153 | 13.739131 | 20.26206 | 0.039441213 | -0.560490101 | Down |
| ENSGALG00000000568 | 1.4262495 | 2.1439254 | 0.034768917 | -0.588028326 | Down |
| ENSGALG00000006394 | 6.8852797 | 10.407685 | 0.049737353 | -0.596062036 | Down |
| ENSGALG00000054424 | 1 | 1.5201987 | 0.009808462 | -0.604259906 | Down |
| ENSGALG00000048371 | 1.309186 | 2.0022547 | 0.019570136 | -0.612955426 | Down |
| ENSGALG00000054422 | 1.0702465 | 1.6520115 | 0.04398421 | -0.626280612 | Down |
| ENSGALG00000010662 | 1.1505 | 1.7881423 | 0.043680854 | -0.636200567 | Down |
| ENSGALG00000012873 | 2.5050106 | 3.8963726 | 0.042678203 | -0.637314937 | Down |
| ENSGALG00000010473 | 9.925732 | 15.587773 | 0.0464343 | -0.65116942 | Down |
| ENSGALG00000053709 | 8.567743 | 13.556805 | 0.04419684 | -0.662030101 | Down |
| ENSGALG00000046697 | 1 | 1.5824717 | 0.020928519 | -0.6621797 | Down |
| ENSGALG00000015234 | 2.2479155 | 3.5606406 | 0.048464082 | -0.663549017 | Down |
| ENSGALG00000008852 | 24.19337 | 38.98015 | 0.006158899 | -0.6881279 | Down |
| ENSGALG00000051506 | 2.18156 | 3.7013004 | 0.041819397 | -0.762672077 | Down |
| ENSGALG00000050696 | 2.86354 | 4.9488387 | 0.033770032 | -0.789290264 | Down |
| ENSGALG00000040959 | 11.339497 | 20.414488 | 0.029750656 | -0.848236739 | Down |
| ENSGALG00000031866 | 1.2963281 | 2.3451083 | 0.03503509 | -0.85522364 | Down |
| ENSGALG00000024871 | 1.4465395 | 3.002732 | 0.042782877 | -1.053669998 | Down |
| ENSGALG00000053923 | 1 | 4.8553557 | 0.00898755 | -2.27957699 | Down |
| ENSGALG00000052069 | 1 | 5.8772297 | 0.006898978 | -2.555136284 | Down |

**Table S5.** List of DEGs in hypothalamus samples between HEP and LEP chickens

| **GeneID** | **Mean-HEP** | | **Mean-LEP** | **Adj p value** | **log2Ratio (LEP/HEP)** | **Type** |
| --- | --- | --- | --- | --- | --- | --- |
| ENSGALG00000043064 | | 47.291756 | 6.047401 | 0.01466133 | 2.96720156 | Up |
| ENSGALG00000026559 | | 72.73335 | 12.566779 | 0.04062372 | 2.533002107 | Up |
| ENSGALG00000013583 | | 7.30046 | 1.5438553 | 0.00919304 | 2.24144983 | Up |
| ENSGALG00000029401 | | 4.4817066 | 1.5495187 | 0.04709496 | 1.532228038 | Up |
| ENSGALG00000015195 | | 383.45673 | 194.0522 | 0.03023937 | 0.982619001 | Up |
| ENSGALG00000037332 | | 268.8034 | 174.62065 | 0.04194763 | 0.622327209 | Up |
| ENSGALG00000003734 | | 22.373316 | 16.812555 | 0.03139392 | 0.41224011 | Up |
| ENSGALG00000052997 | | 1.0602856 | 1 | 0.00227538 | 0.084452923 | Up |
| ENSGALG00000034337 | | 41.667046 | 75.67159 | 0.03161161 | -0.860844936 | Down |
| ENSGALG00000017246 | | 1.96341 | 16.063276 | 0.03279482 | -3.032332778 | Down |

**Table S6. GO annotation of differential expression genes in high and low egg production in Pituitary gland**

| **Term** | **Category** | **Description** | **LogP** | **Symbols** |
| --- | --- | --- | --- | --- |
| GO:0009314 | BP | response to radiation | -8.06133838 | CBL,CDS1,CREBBP,ECT2,FANCG,XRCC6,GRIN1,HMGCR,HMGCS1,PPEF1,XRCC5,NR2E3,NOX4,GRK7,SDE2 |
| GO:0032481 | BP | positive regulation of type I interferon production | -7.413098774 | CREBBP,XRCC6,NFKB1,POLR2E,XRCC5,FANCG,MPG,SDE2,HACD1,APLF,ECT2,OXSR1,CBL,ACSL1,HMGCR,RAP2B,FGFR3,PRKCZ,VAV3,NOX4,PPP1R9A,ALKAL2,DPYSL2,EPHA7,SOS1,TACC3 |
| GO:1902017 | BP | regulation of cilium assembly | -6.74508663 | EVI5,TBC1D8,ATMIN,TBC1D19,TBC1D22B,CENPJ,TBC1D24,PPP1R9A,PCM1,RAP2B,VAV3,SPATA6,CFAP221,CRMP1,DPYSL2,EPHA7,GRIN1,SPOCK1,ALKAL2,DNAH1,SNX10,TTC26,CFAP126,GMNC,ECT2,SOS1,UNC13B,XPO7,LAMTOR1,RAB11FIP1,TAX1BP3 |
| GO:0098813 | BP | nuclear chromosome segregation | -6.105513829 | BUB1B,ECT2,GEM,ZW10,TACC3,CEP55,CENPJ,CENPU,CENPW |
| GO:0044458 | BP | motile cilium assembly | -5.293136985 | ATMIN,SPATA6,CENPJ,CFAP221 |
| GO:0050730 | BP | regulation of peptidyl-tyrosine phosphorylation | -5.200100296 | CBL,EPHA7,FGFR3,IL15,PRKCZ,RAP2B,NOX4,SOCS4,ALKAL2 |
| GO:0001678 | BP | cellular glucose homeostasis | -4.886478652 | HMGCR,FOXK2,SRI,UNC13B,GJB6,NOX4,AACS,HNF1A,GEM,HADH,NFKB1,OXTR,OXSR1,PPP1R9A,RAB11FIP1,FAM3D,ECT2,PCM1,PRKCZ,CENPJ,CNST |
| GO:0009112 | BP | nucleobase metabolic process | -4.71654881 | CRMP1,DPYSL2,GMPS |
| GO:1903510 | BP | mucopolysaccharide metabolic process | -4.644400731 | IL15,NFKB1,UGDH,B4GALT4,LYVE1,CHSY3,ACSL1,FOXK2,TK2,GMPS,HACD1,FUT9,GAL3ST2 |
| GO:0007265 | BP | Ras protein signal transduction | -3.329700968 | CBL,CDK2,DOK1,ECT2,RAP2B,RRAS,SOS1,VAV3,TAX1BP3,LAMP1,SH3BP2,VAPA,LSM4,PPP1R9A,CENPJ |
| GO:0007584 | BP | response to nutrient | -3.239689267 | ASCL1,NQO1,ACSL1,GRIN1,HMGCR,HMGCS1,IL15,AACS,BRIP1,BNIP3,CBL,ECT2,KCNC1,NFKB1,OXTR,ADAM9,TXNRD2,PPP1R9A,FOXK2,ZFYVE1,WDR24 |
| GO:1901293 | BP | nucleoside phosphate biosynthetic process | -2.88089512 | CDS1,ACSL1,GMPS,HACD1,ATP5MC1,HMGCS1,FOXK2,SLC4A1,TK2,LPIN1,PIK3R5,PIGN,PNPO,LHPP,NAXE |
| GO:0045936 | BP | negative regulation of phosphate metabolic process | -2.804271236 | CBL,FKTN,HMGCR,IGFBP3,PKIA,PRKCZ,DNAJC3,CTDSPL,ZFYVE1,SNX25,FAM122A,SOCS4,CNST,SPRED2,GRXCR1 |
| GO:0032580 | CC | Golgi cisterna membrane | -5.134453172 | B4GALT4,FUT9,MOB4,TMEM87A,GAL3ST2,CHSY3 |
| GO:0031594 | CC | neuromuscular junction | -4.37689699 | EPHA7,SPOCK1,UNC13B,PPP1R9A,TBC1D24,GRIK4,GRIN1,PRKCZ,SOS1,ANKS1B,CBL |
| GO:0033267 | CC | axon part | -3.845358949 | CBL,GRIN1,PRKCZ,SPOCK1,SRI,UNC13B,AP3M2,PPP1R9A,TBC1D24,KCNC1,FSTL3 |
| GO:0098686 | CC | hippocampal mossy fiber to CA3 synapse | -3.173330038 | EPHA7,GRIK4,UNC13B,GRIN1,KCNC1,SHISA9,KCNJ1,KCNS3,CACHD1 |
| GO:0005815 | CC | microtubule organizing center | -2.89306588 | BUB1B,CDK2,CRMP1,PCM1,PRKCZ,EVI5,DLGAP5,TACC3,CEP55,CENPJ,CENPU |
| GO:0016405 | MF | CoA-ligase activity | -3.522559754 | ACSL1,SLC27A6,AACS |
| GO:0050662 | MF | coenzyme binding | -2.895863132 | GAD2,HADH,HMGCR,UGDH,TXNRD2,NOX4,PNPO,ZC3HAV1,OGFOD3,PHYKPL,FLVCR1,BRIP1,COQ10A |

**BC:** Biological Processes; **MF:** Molecular Functions; **CC:** Cellular Components

**Table S7. GO annotation of differential expression genes in high and low egg production in Ovary**

| **Term** | **Category** | | **Description** | **LogP** | **Symbols** | |
| --- | --- | --- | --- | --- | --- | --- |
| GO:1901361 | | BP | organic cyclic compound catabolic process | -8.26415 | GAPDH,EIF3E,PRKCD,RPL7,RPL12,RPL30,RPL27A,RPL34,TDO2,FGF23,KYNU,EIF3I,BBS2,GOLGA4,PREPL,RAB8B,PARD3,HDAC3,RELN,SH3GLB1,DECR2,GNPTAB,DNAJC19,ZDHHC15 |  |
| GO:0031647 | | BP | regulation of protein stability | -5.49762 | EPHA4,GAPDH,PRKCD,STK3,USP7,HDAC3,CSNK1A1,EIF3E,MEF2C,POU4F2,PLCL2,DTNBP1,DAB2IP,LGR5,LBX1 |  |
| GO:0048667 | | BP | cell morphogenesis involved in neuron differentiation | -5.37116 | CTTN,EPHA4,GDNF,GOLGA4,MEF2C,POU4F2,RELN,CLRN1,PARD3,LGR6,ISL2,DTNBP1,C8orf37,GABRB2,HTR1F,OPRM1,PREPL,NLGN4Y,PLCL2,TRPM1 |  |
| GO:0035520 | | BP | monoubiquitinated protein deubiquitination | -4.90969 | USP7,USP15,MYSM1,ADAM11,RELN,PSMA1,PREPL,FGL2,ERAP1,USP53,OSGEPL1,SPPL2A |  |
| GO:0071417 | | BP | cellular response to organonitrogen compound | -4.60465 | AANAT,EPHA4,GABRB2,GH1,MEF2C,OPRM1,POU4F2,PRKCD,AHCYL1,ATP6V0A2,RAB8B,CPEB2,BBS2,FGF23,LGR5,SRSF6 |  |
| GO:0032409 | | BP | regulation of transporter activity | -4.28509 | MEF2C,OPRM1,PRKCD,AHCYL1,THADA,FGF23,TNFSF13B,PLCL2 |  |
| GO:0006586 | | BP | indolalkylamine metabolic process | -4.16693 | AANAT,TDO2,KYNU,GFPT2,AHCYL1,PAH,PSMA1,AIMP2,PHYKPL,GALK2,GAPDH,FGF23,DERA |  |
| GO:0021517 | | BP | ventral spinal cord development | -4.09313 | RELN,LBX1,ISL2,FOXN4,GDNF,EPHA4 |  |
| GO:0043473 | | BP | pigmentation | -3.97075 | BBS2,MEF2C,PMEL,DTNBP1,MYSM1 |  |
| GO:0048568 | | BP | embryonic organ development | -3.66496 | GDNF,HOXD9,MEF2C,STK3,CLRN1,IRX5,LBX1,GTPBP3,FOXN4 |  |
| GO:0003407 | | BP | neural retina development | -3.65797 | GNAT2,POU4F2,IRX5,FOXN4,EPHA4,GABRB2,TRPM1,CLRN1,LGR5,TRIP11,C8orf37,BBS2,PRPH2,DNAJC19 |  |
| GO:0048638 | | BP | regulation of developmental growth | -3.58366 | BBS2,CTTN,GH1,GOLGA4,MEF2C,POU4F2,MAPK11,STK3,PREPL |  |
| GO:0006400 | | BP | tRNA modification | -3.58065 | TRDMT1,THADA,OSGEPL1,GTPBP3 |  |
| GO:0014066 | | BP | regulation of phosphatidylinositol 3-kinase signaling | -3.4928 | GH1,PPP2R5C,RELN,DAB2IP,DIPK2A,EPHA4,USP7,TRIM39 |  |
| GO:0002181 | | BP | cytoplasmic translation | -3.38801 | EIF3E,RPL30,EIF3I |  |
| GO:0060828 | | BP | regulation of canonical Wnt signaling pathway | -3.34971 | CSNK1A1,PSMA1,STK3,LGR5,LGR6,PRDM15,DAB2IP |  |
| GO:0007422 | | BP | peripheral nervous system development | -3.34038 | GDNF,HOXD9,PARD3,ISL2,BBS2,EPHA4,OPRM1 |  |
| GO:0032588 | | CC | trans-Golgi network membrane | -5.02571 | LGR5,RAB9A,COG5,LGR6,GOLGA4,GALNT2,TRIP11,SH3GLB1,CSGALNACT2,GNPTAB,SCAMP4,DIPK2A |  |
| GO:0016604 | | CC | nuclear body | -4.05782 | CSNK1A1,EIF3E,MEF2C,POU4F2,SRSF6,USP7,API5,TRIP11,TELO2,SDCBP2,RAB8B,THAP1,PRDM15 |  |
| GO:0016769 | | MF | transferase activity, transferring nitrogenous groups | -3.76027 | GAPDH,GFPT2,PHYKPL |  |

**BC:** Biological Processes; **MF:** Molecular Functions; **CC:** Cellular Components


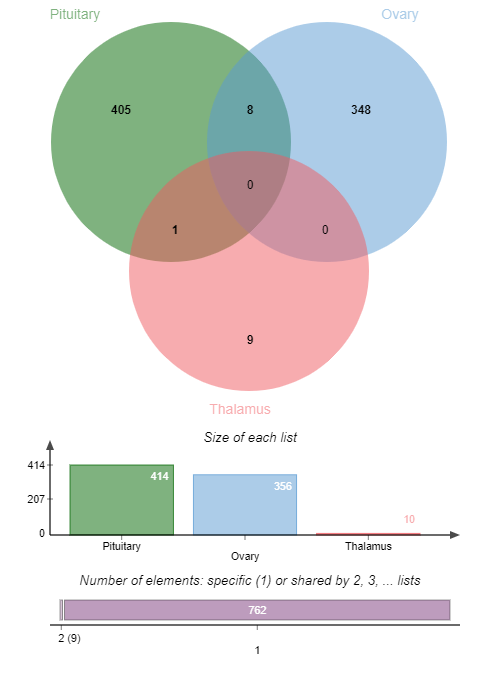


Figure S1. Venn diagram display shared significant DEGS among pituitary, ovary and hypothalamus of HEP and LEP chickens.
